# H_2_S biosynthesis and catabolism: new insights from molecular studies

**DOI:** 10.1007/s00018-016-2406-8

**Published:** 2016-11-14

**Authors:** Peter Rose, Philip K. Moore, Yi Zhun Zhu

**Affiliations:** 1 School of Life Science, University of Lincoln, Brayford Pool, Lincoln, Lincolnshire, LN6 7TS UK; 2 State Key Laboratory of Quality Research in Chinese Medicine and School of Pharmacy, Macau University of Science and Technology, Macau, China; 3 Department of Pharmacology, National University of Singapore, Lee Kong Chian Wing, UHL #05-02R, 21 Lower Kent Ridge Road, Singapore, 119077 Singapore

**Keywords:** Hydrogen sulfide, Biosynthesis, Catabolism, Molecular models

## Abstract

Hydrogen sulfide (H_2_S) has profound biological effects within living organisms and is now increasingly being considered alongside other gaseous signalling molecules, such as nitric oxide (NO) and carbon monoxide (CO). Conventional use of pharmacological and molecular approaches has spawned a rapidly growing research field that has identified H_2_S as playing a functional role in cell-signalling and post-translational modifications. Recently, a number of laboratories have reported the use of siRNA methodologies and genetic mouse models to mimic the loss of function of genes involved in the biosynthesis and degradation of H_2_S within tissues. Studies utilising these systems are revealing new insights into the biology of H_2_S within the cardiovascular system, inflammatory disease, and in cell signalling. In light of this work, the current review will describe recent advances in H_2_S research made possible by the use of molecular approaches and genetic mouse models with perturbed capacities to generate or detoxify physiological levels of H_2_S gas within tissues.

## Introduction

Hydrogen sulfide (H_2_S) has gained acceptance by researchers, as the third gaseous mediator identified in mammals alongside nitric oxide (NO) and carbon monoxide (CO). Over the past decade, this molecule has been shown to be synthesised by a range of tissues in which it functions as a signalling molecule with distinct physiological and biochemical effects [1–3]. To date, the spectrum of signalling systems identified include, but is not restricted to, nuclear factor-kappa beta (NF-κB), the activity of several kinases, including p38 mitogen-activated protein kinase (p38 MAPK) [4], c-JunNH_2_-terminal kinase (JNK) [5], extracellular signal-regulated kinase (ERK) [6], phosphoinositide 3-kinase-protein kinase B (PI-3K-Akt) [7], protein kinase C (PKC) [8], nuclear factor erythroid 2-related factor 2 (Nrf-2) [9], p53 [10], AMP-activated protein kinase [11], proliferator-activated receptor γ [12], NAD-dependent deacetylase sirtuin-1 (SIRT1) [13], SIRT3 [14], and mechanistic target of rapamycin (mTOR) [15]. Studies focused on delineating these molecular networks have revealed H_2_S to have important roles in cytoprotection [16–20], inflammation [21–24], vascular function [25–27], neurological systems [28], tissue repair and healing [29–34], apoptosis and the cell cycle [35, 36], mitochondrial function and energy metabolism and biogenesis [37–48], obesity [49–53], and in ageing [54–60]. What function H_2_S which plays in these processes ranges from its ability to act as an antioxidant during episodes of elevated free-radical production [61, 62] to direct post-transcriptional modification of cellular proteins via S-sulfhydration [63, 64]. In practise, the signalling effects of H_2_S are more complex due to the fact that this gas readily interacts with other signalling molecules, such as reactive oxygen and nitric-oxide species [65–67]. Aside from enzymatic routes of synthesis, recent evidence has also shown indirect or secondary sites of H_2_S production. These sites include the endogenous liberation from persulfides and polysulfide species, both endogenous and dietary derived, along with bacterial sources present within the gastrointestinal tract [68–79]. How these pools of H_2_S are coordinated within localised, as well as distal sites, and how these systems influence disease pathology and longevity in mammals is one of the key questions currently being explored by researchers in this field.

## H_2_S biosynthesis and catabolism

**Fig. 1:**
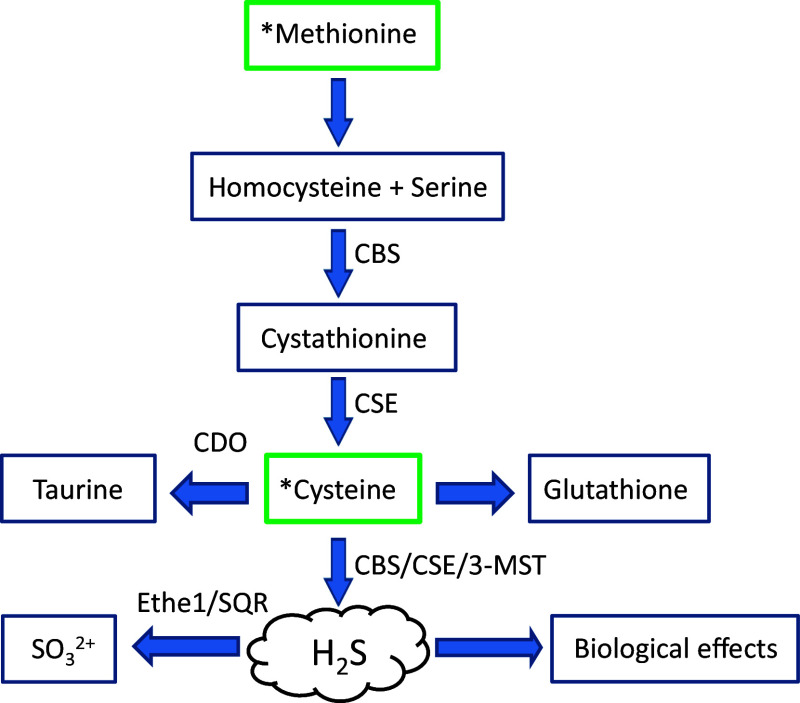
Generalised overview of H_2_S production and degradation within mammalian tissues. The dietary amino acids, methionine and cysteine, serve as the primary substrates for the trans-sulfuration pathway and in the production of H_2_S. The levels of H_2_S within cells and tissues will be governed by the rates of synthesis by the enzymes cystathionine β synthase (CBS, EC 4.2.1.22), cystathionine-γ-lyase (CSE, EC 4.4.1.1), 3-mercaptopyruvate sulfurtransferase (3-MST, EC 2.8.1.2), versus the rates of oxidation and detoxification by the enzymes ethylmalonic encephalopathy protein 1 (ETHE1, EC: 1.13.11.18) and sulfur:quinone oxidoreductase (SQR, EC 1.8.5.4). Alternatively, the levels of the substrate cysteine may be depleted via the catabolic actions of cysteine dioxygenase (CDO, EC: 1.13.11.20)

Biosynthetic and degradative pathways involved in H_2_S production and consumption are largely mediated by cystathionine β synthase (CBS, EC 4.2.1.22), cystathionine-γ-lyase (CSE, EC 4.4.1.1), 3-mercaptopyruvate sulfurtransferase (3-MST, EC 2.8.1.2), ethylmalonic encephalopathy protein 1 (ETHE1, EC: 1.13.11.18), mitochondrial sulfide–quinone oxidoreductase (SQR, EC 1.8.5.4), and cysteine dioxygenase (CDO, EC: 1.13.11.20) (Fig. 1). Biochemical and pharmacological aspects relating to these enzymatic systems have recently been covered in great detail [80, 81] and will, therefore, only be touched upon herein. Moreover, whilst the roles of ETHE1, SQR, and CDO may not appear obvious at first sight, their potential influence on H_2_S tissue levels, via catabolic effects on either H_2_S directly or on the amino-acid cysteine justifies inclusion. Since the potential importance of these enzymes has, until now, been largely ignored, we believe that some discussion is warranted, if only at the very least, to stimulate debate and hopefully encourage future studies using the available murine genetic knockout models. Furthermore, the possibility of the existence of polymorphisms linked to genes encoding H_2_S detoxification enzymes is intriguing. How such variants influence tissue H_2_S turnover rates and physiological effects remains largely unexplored. Thus, the expression levels and catabolic effects of each of these enzymes may well influence exposure levels of cells, tissues, and organs to this biologically active gas. It is for this reason that these systems will be described across physiologically relevant models, including the mouse, *Mus musculus,* and to a lesser extent in *Caenorhabditis elegans*, *Drosophila melanogaster,* and *Danio rerio*. Collectively, these models will pave the way to a better understanding of the biological significance of this gaseous molecule and could potentially assist in the development of future pharmacologically active entities. The review will also address some of the recent findings relating to H_2_S biology in which genetic approaches, including gene knockdown and genetic model systems, have been employed to explore the functional role of this gas.

## Pharmacological approaches to manipulate H_2_S levels within biological systems

**Fig. 2:**
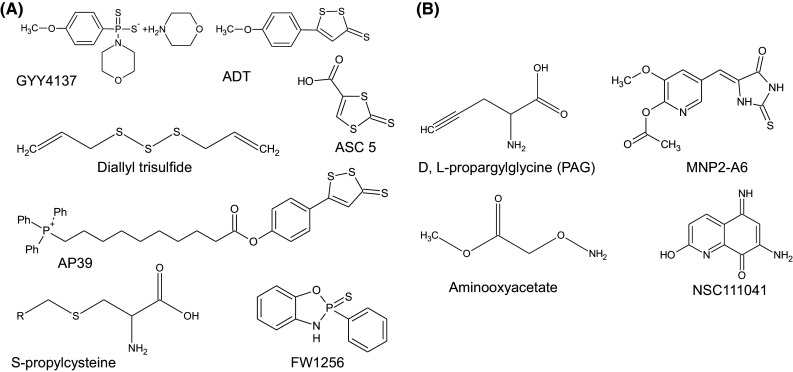
Over the last 10 years, a wide range of H_2_S donor molecules have been developed to assist in determining the biological effects of H_2_S under differing physiological and pathophysiological states. **a** H_2_S donor molecules commonly used experimentally as research tools to manipulate cellular levels of H_2_S gas. **b** Structures of several inhibitor molecules that target CBS and CSE

In general, our current understanding of H_2_S biology has arisen from work focused on enzymes of the trans-sulfuration pathway. For detailed coverage of the biochemical aspects relating to these enzymatic systems, we refer interested readers elsewhere [82–84]. By and large, the maintenance of the cellular H_2_S homeostatic equilibrium is governed by a small group of enzymes that are involved in the catabolism of the amino-acid cysteine, namely, CBS, CSE, and 3-MST. Both CBS and CSE appear to be the major enzymatic routes for the production of H_2_S within biological systems. Tissue specific expression of CBS predominates in the brain, nervous system, liver, and kidney, while CSE is expressed in the liver and in vascular and non-vascular smooth muscle. However, recent studies have reported on the expression of CBS in HUAEC cells, the uterine artery, mesenteric artery, and carotid body [85]. Furthermore, the expression of CBS in the uterine artery was found to be stimulated at the hormonal level [86]. This finding suggests a critical role for H_2_S within the reproductive tract. 3-MST is localised to mitochondria and produces H_2_S in a coupled reaction with the enzyme cysteine aminotransferase [87]. Information on the degradative and detoxification routes for H_2_S within biological systems is less widely reported. What is known is that the degradation or loss of tissue H_2_S appears to occur via a number of distinct pathways that likely working in concert. For example, chemical processes, such as (1) the direct oxidation of H_2_S to thiosulfate in the presence of O_2_ and transition metals or (2) via enzymatic processes that include SQR and ETHE1 systems [88–91]. Functional roles for the enzymes rhodanese (EC 2.8.1.1) and sulfite oxidase (EC 1.8.3.1) have also been proposed, yet data are currently lacking for these detoxification routes [92–95]. For many studies, manipulation of cellular and tissue levels of H_2_S is required and historically, this has been achieved utilising inhibitor and/or donor molecules targeting the H_2_S biosynthetic pathway (Fig. 2). The widely used CSE inhibitor, dl-proparylglycine, for example, can increase disease severity in animal models of colitis [96], myocardial ischemia–reperfusion-induced injury [97], and also has anti-hyperalgesic effects [98] and has reported inflammatory as well as anti-inflammatory effects in rodent models [21]. These studies indicate that the inhibition of H_2_S biosynthetic enzymes, and therefore, the production of H_2_S within tissues and cells typically leads to increased disease severity which effects are reversed by the use of H_2_S donor molecules. To date, several pharmacological inhibitors are now available for use in this field, including hydroxylamine (HA), trifluoroalanine, aminooxyacetate (AOAA) (for CBS), and d,l-propargylglycine (PAG) or β-cyanoalanine (BCA) (for CSE), that have provided a means to manipulate tissue H_2_S levels [99–103]. Other newer inhibitory molecules with greater specificity and enhanced potency have also been characterized, but sadly, many of these are not currently commercially available. For instance, in the work of Thorson, a marine invertebrate compound library consisting of 160 characterized marine natural products and 80 purified synthetic derivatives aided in the identification of several small molecular weight inhibitors of CBS with IC_50_ values below 200 μM (range 83–187 μM) [104, 105]. So far, a number of similar library-based screening approaches have proven fruitful in the identification of novel inhibitory molecules targeting CSE, CBS, and/or both. Indeed, Zhou and colleagues have utilised a tandem well-plate screening system to assess potential inhibitory molecules that target CSE and CBS. This approach involved screening 21599 chemical entities that lead to the identification of several potent inhibitory molecules designated NSC111041, NSC67078, and SP14311008 [106]. Interestingly, NSC111041 and SP14311008 appear to target these enzymes at sites distal to the PLP binding site. This finding could perhaps serve to assist in the development of new classes of inhibitory molecules. Lastly, the pharmacological targeting of 3-mercaptopyruvate sulfotransferase is less widely reported, however, several inhibitor molecules have been identified base on their abilities to affect the rate of enzyme catalyzed thiocyanate formation in vitro. This structurally diverse class of inhibitor molecule includes hypotaurine, methanesulfinic acid along with pyruvate, phenylpyruvate, oxobutyrate, and oxoglutarate [107]. These molecules appear to inhibit 3-MST in a concentration-dependent manner and have been determined to be uncompetitive inhibitors of 3-MST with respect to 3-mercaptopyruvate [108, 109]. Typical IC_50_ values for all three alpha-keto acids ranging between 9.5 and 13.7 mM. In spite of this information, no direct confirmation of their inhibitory action towards 3-MST and it ability to generate H_2_S has been reported.

## Genetic evidence for a role of CBS, CSE, and 3-MST in health and disease

The established roles for CBS, CSE in sulfur amino-acid metabolism are widely recognised [110–112] and it is of interest that a number of polymorphisms in the genes coding for these proteins are linked to a range of pathophysiological conditions in humans [113, 114]. For example, there are an estimated 150 mutations in the CBS locus and of these approximately 20 appear to have altered enzymatic activity [115]. A consequence for this loss often being homocystinuria [116]. Interestingly, the CBS T833C variant has been associated with premature coronary artery disease [117], essential hypertension [118], and an increased risk of stroke [119]. Similarly, the CBS 844ins68 polymorphism is linked to increase risk of breast cancer [120], spontaneous cervical artery dissections [121], raised plasma homocysteine levels [122], and elevated homocysteine–thiolactone concentrations [123]. Homocysteine–thiolactone is pro-atherogenic [124, 125], and can promote optic lens dislocation [126]. Of equal interest, are polymorphisms linked to the CSE gene that predispose individuals to hypertension [127] and in some cases raised plasma homocysteine levels [128]. Several of these polymorphisms have been described in patients with cystathioninuria, and a single nucleotide polymorphism in CSE, c.1364G>T, is linked to elevated plasma homocysteine levels [128]. The influence of the rs1021737 and rs482843 CSE polymorphisms in preeclampsia has been raised [129], and a proposed role in the development of chronic hypertension reported [111]. Importantly, many of these polymorphic variants have reduced *V*_max_ for the substrate cystathionine [130]. Polymorphisms linked to the 3-MST gene are also known and the recent characterisation of a nonsense mutation (Tyr85Stop) that leads to the production of a severely truncated protein lacking enzymatic activity has been described [131]. In spite of the information relating to H_2_S biosynthetic enzymes, data are currently lacking as to whether these polymorphic variants influence H_2_S biosynthetic rates. However, supporting evidence would indicate that this may be the case. Research utilising site-directed mutagenesis studies of the CBS protein has identified several key cysteine residues that are directly involved in the regulation of basal CBS activity and in H_2_S production [132], and changes in the CBS binding site of the allosteric activator S-adenosylmethionine reduce H_2_S synthesis by this enzyme [133]. Similarly, several amino-acid residues in CSE have been identified that are actively involved in H_2_S production [134]. Therefore, the possibility that known polymorphisms for CBS, CSE, and 3-MST would influence enzymatic activity of these proteins, and therefore, tissue H_2_S levels is not unreasonable.

Further circumstantial evidence linking impaired tissue biosynthesis rates of H_2_S and disease are provided from a range of additional sources. Loss of function in either CBS or CSE can increase the risk of individual developing cardiovascular diseases. Moreover, decreased H_2_S production rates in mice predispose animals to vascular remodeling, hypertension, and early the development of atherosclerosis. Therefore, the idea that H_2_S may have an important function within the cardiovascular system and at other sites is not a new concept. Indeed, H_2_S and allied donor drugs can reduce homocysteine mediate cellular stress responses and tissue damage in mammalian systems [135–139]. In addition, it is widely recognised that H_2_S can directly affect blood pressure, alter lipid metabolism, inhibit monocytes adhesion and activate the endothelium [140, 141], promote vasorelaxation [142], and induce angiogenesis [143]. H_2_S also mediates vascular smooth muscle cell proliferation, migration, and apoptosis [144–146], inhibits macrophage foam cell formation [147], chemotaxis [148], and inflammation [23, 149], and decreases vascular calcification [150], platelet aggregation, and thrombogenesis [151, 152] (reviewed in [153, 154]). Importantly, in humans, decreased plasma H_2_S concentrations are found to correlate with the activation of protein kinase CβII in uremic accelerated atherosclerosis patients [155] and in chronic haemodialysis patients with diabetic nephropathy [156]. Diminished levels of plasma H_2_S are also reported to be significantly lowered in CHD patients and in smokers as compared to normal subjects [157], in essential hypertensive children suffering from a metabolic imbalance of homocysteine and hydrogen sulfide [158], and are decreased in patients on chronic haemodialysis due to reduced CSE expression [159]. Lower H_2_S levels also correlate with the accumulation of lanthionine in the blood of uremic patients [160]. These changes potentially contribute to hyperhomocysteinemia in uraemia. Intriguingly, homocysteine has been reported to decrease H_2_S production in macrophages by increasing promoter DNA methylation and transcriptional repression of CSE [161]. In addition, the cardioprotective effects of atorvastatin appear to be partly mediated by the effects of this drug on the expression of CSE and associated increases in the generation of H_2_S [162]. Therefore, from the available evidence, it is clear that multiple pathologies and mechanisms underpin these diseases, but, intriguingly, a lack of H_2_S production seems to be at least one common thread. For this reason, the characterisation of gene polymorphisms linked with enzymes associated with H_2_S synthesis and its degradation requires further exploration. This could provide a greater understanding of how such polymorphisms influence enzymatic function and this may, in the future, be found to translate to changes in circulatory H_2_S levels. A key question is how do changes in the expression levels of enzymes involved in H_2_S homeostatic regulation, and their associated mutations cause disease and what are the molecular mechanisms responsible for this? To answer these questions, new approaches that include genetic models of H_2_S deficiency and/or overproduction have been adopted. Specifically, knockout animals lacking genes encoding for CSE, CBS, 3-MST, CDO and ETHE1. In the case of studies utilising these models, a greater understanding of how H_2_S functions as a signalling molecule and how this translates to influencing physiological and biochemical processes in vivo is pushing the boundaries of our current views for this gas. Importantly, findings from such work may provide routes for patient screening prior to pharmacological intervention with H_2_S releasing drugs to restore H_2_S levels.

## Molecular approaches to alter H_2_S biosynthetic capacity in cells and animals

In addition to pharmacological approaches to alter tissue H_2_S concentrations, a number of researchers have adopted siRNA methodologies to assist in loss of function studies by targeting H_2_S biosynthetic enzyme expression levels. These techniques have been particularly amenable for use in cell-culture systems. As shown in Table 1, these approaches have assisted researchers in the manipulation of the expression levels of enzymes involved in H_2_S homeostatic regulation across a range of cell types. These technologies, while technically more challenging, have shown that H_2_S is involved in cellular proliferation and apoptosis [146], endoplasmic reticulum stress, and insulin secretion [176], and NF-κB and MAP kinase signalling and inflammation in macrophages [166, 167]. Curiously, the silencing of 3-MST has revealed this enzyme to be involved in the H_2_S production that in turn supports mitochondrial bioenergetics [39, 40]. Currently, siRNA and shRNA systems targeting CSE and CBS can be obtained from a range of commercial suppliers, including, but not exclusively by, CAYMAN chemicals, Addgene (Cambridge, MA, USA), and Santa Cruz Biotechnology (Texas, USA) or can be custom synthesised by IDT DNA technologies (Glasgow, UK).

**Table 1:** Studies utilising cell-culture models to explore the loss of function or overexpression of H2S synthesising enzymes

Disease model	Transgenic system	Cell type	Consequence	References
Cystathionine gamma lyase				
CVD	CSE adenovirus gene transfer	Stably CSE overexpression in HEK-293 cells	Increases in CSE mRNA levels, CSE proteins, leading to increased intracellular production rates of H_2_S. This correlated with the inhibition of cell proliferation and DNA synthesis. Sustained ERK activation and upregulation of the cyclin-dependent kinase inhibitor p21Cip/WAK^−1^ was also noted	[163]
	CSE adenovirus gene transfer	Stably CSE overexpression in Human aorta smooth muscle cells	Increase in the expression of CSE protein and a committed increase in H_2_S production rates. Cell growth inhibition and the induction of apoptosis noted in CSE overexpressing cells. Apoptosis was associated with an increased in ERK and p38 MAPK activation, upregulation of p21(Cip/WAK-1), and downregulation of cyclin D1 expression. Inhibiting endogenous background CSE gene expression, and direct administration of H_2_S at 100 microM induced apoptosis in HASMCs	[146]
	Transfected with miR-30 mimics	HEK293 cells and primary neonatal rat myocardial cells	Overexpression of miR-30 family members decreases the expression of CSE protein and H_2_S production. Reduced CSE expression sensitised cells to hypoxic conditions. Overexpression of CSE was cytoprotective in this model. Knockdown of miR-30 family members leads to the upregulation of CSE and H_2_S production rates	[164]
Diabetes	CSE adenovirus gene transfer	Transfection of insulin secreting beta cell line INS-1E cells	CSE overexpression stimulates INS-1E cell apoptosis via increased endogenous production of H_2_S. Ad-CSE transfection inhibited ERK1/2 but activated p38 MAPK. Overexpression of CSE or H_2_S treatment increased BiP and CHOP levels indicators of endoplasmic reticulum (ER) stress	[176]
Inflammation	siRNA targeting mouse CSE	Murine Raw264.7 macrophages and primary macrophage isolated from adult male C57BL/6 mice	CSE overexpression reduced the ox-LDL-stimulated tumor necrosis factor-α (TNF-α) generation in Raw264.7 and primary macrophage while CSE knockdown enhanced it	[149]
	siRNA targeting mouse CSE	Human chondrocytes and mesenchymal progenitor cells	CBS- and CSE-siRNA treatment sensitises cells to oxidative stress leading to loss of cell viability as determined using the MTT assay. l-cysteine, a substrate for CSE and CBS, fails to protect against SIN-1, H_2_O_2_ and 4-HNE induced cell death in chondrocytes in silenced cells	[165]
	siRNA targeting mouse CSE	Murine RAW 264.7 macrophages	Lipopolysaccharide (LPS) treatment of RAW 264.7 cells promotes increased CSE mRNA and protein levels along with increased production of proinflammatory cytokines (TNF-α, IL-1β, IL-6, and MCP-1) and nitric oxide (NO). Silencing of CSE reduced proinflammatory mediator levels and enhanced NO production	[166]
	siRNA targeting mouse CSE	Murine RAW 264.7 macrophages	CSE silencing reduced inflammation status by attenuating the activity of NF-κB in lipopolysaccharide- (LPS-) stimulated macrophages. Reduced production of inflammatory mediators via inhibition of extra cellular signal-regulated kinase 1/2 (ERK1/2) phosphorylation	[167]
Preeclampsia	siRNA targeting mouse CSE and adenovirus gene transfer	Human umbilical vein endothelial cells (HUVEC)	Downregulation of CSE results in an increased release of soluble fms-like tyrosine kinase-1 (sFlt-1) and soluble endoglin (sEng); both proteins involved in angiogenesis. Overexpression of CSE results in the inhibition of sFlt-1 and sEng release	[168]
Osteoporosis	siRNA targeting mouse CSE	Bone marrow mesenchymal stem cells (BMMSCs)	Knockdown of CSE lead to increased cell proliferation, reduced capacity for forming mineralized nodules in vitro, and downregulation of Runx2 and ALP. Reduction of H_2_S levels resulted in a cascade response in BMMSCs, including altered Ca^2+^ channel sulfhydration, Ca^2+^ influx, Wnt/β-catenin signaling, and osteogenic differentiation	[169]
	siRNA targeting mouse CSE	Murine RAW 264.7 macrophages	CSE silencing inhibited osteoclast formation by reducing the expression of the typical osteoclast markers, Cathepsin K, TRAP and MMP9	[229]

Disease model	Transgenic system	Model system	Consequence	References
Cystathionine-beta synthetase				
CVD	Transfected with CBS cDNA subcloned into the plasmid pcDNA3	Mouse aortic endothelial cells (MAEC)	Transfection of endothelial cells with cystathionine-beta-synthase (CBS) reduced Hcy accumulation in high methionine-fed cells. Reduced inflammatory response, as evident by attenuated ICAM-1 and VCAM-1 expression and reduced expression of collagen type-1 expression and MMP-9 activity	[170]
	Lentiviral CBS-targeting short hairpin RNA (shRNA)	Human umbilical vein endothelial cells (HUVEC) and human aortic endothelial cells (HAEC)	CBS knockdown reduced cell proliferation in both HUVEC and HAEC cells. Expression of p21^WAF-1^ and γ-H_2_AX, both molecular markers of senescence, were induced along with positive staining for β-galactosidase (SA-β-gal). Loss of CBS induces premature endothelial cell senescence	[171]
Cancer	siRNA targeting mouse CBS	A2780, A2780/CP-70, OV202 and SKOV3 human ovarian carcinoma cells	Ovarian cancer cell proliferation was decrease upon CBS silencing as determined via (^3^H)-thymidine incorporation. In CBS silenced A2780 cells cellular ROS levels increase and glutathione levels significantly decrease. Expression of p53 is also induced in A2780 cells with the RelA/p65 subunit of NF-κB showing decreased expression	[172]
	siRNA targeting mouse CBS	Human colonic epithelial cancer cell line HCT116	Silencing lead to a reduction of CBS expression and associated reductions in H_2_S production and cell proliferation. Reduction in ATP synthesis, basal cellular respiration and spare respiratory capacity. A significant reduction in the density of CD31-positive blood vessels within tumour tissue and an increase in vessel branching. Reduced glycolytic functions, possibly due to inhibition of GAPDH activity	[173]
	siRNA targeting mouse CBS	Transfection of immortalized Jurkat human T-lymphocyte cells	Reduced expression of CBS mRNA significantly impaired both CD69 and IL-2 expression in TCR-activated T cells and resulted in decreased adhesion, which could be partially rescued by the addition of exogenous H_2_S	[174]
	Lentiviral CBS-targeting short hairpin RNA (shRNA)	Human breast cancer cells MCF7 and MDA-MB-468	Silencing of CBS increased the steady state levels of adducts 4-HNE and MDA protein in HBC cells. When co-cultured with activated macrophages, HBC cell growth was compromised by CBS silencing	[175]
3-Mercaptopyruvate sulfurtransferase				
Bioenergetics		Mitochondrial Bioenergetics; isolated mitochondria and hepa1c1c7 cells	3-MST mediated stimulation of H_2_S production is reduced in siRNA and shRNA mediated KO of 3-MST. Loss of a functional 3-MST protein correlated with reduced cellular bioenergetics in hepatoma cells, reduced oxygen consumption and H_2_S production rates	[40]
CVD	p-plasmid cytomegaloviral promoter DNA vector system (pcDNA/GFP)	Mouse aortic endothelial cells	MAEC cells transfected with CSE, CBS, MST or gene triplicate. 3-MST overexpression localised to mitochondria with 3MST-transfected cells produced increased amount of H_2_S compared with nontransfected cells. Mitigates Hcy-induced mitophagy and ROS production	[137]

## In vivo knockout models of H_2_S research

Over the last two decades, much has been learnt regarding the biological roles ascribed to H_2_S, yet many questions still remain to be answered. Indeed, little is known regarding the compensatory mechanisms that may exist to maintain physiological levels of H_2_S nor the interplay between biosynthetic routes and the recently characterised detoxification pathways involving ETHE1 and SQR. Establishing links between these two metabolic processes will be important in the future developed of pharmacologically active drugs and inhibitor molecules that target the H_2_S system. The possibility that inhibitors targeting ETHE1 or SQR could offer an alternate means to manipulate H_2_S levels is intriguing. These approaches will most certainly require work within whole physiological systems and perhaps in this instance in the use of transgenic mouse models in which genes encoding for H_2_S synthesising enzymes have been manipulated. Of relevance here then are the approaches taken to generate mice devoid of H_2_S biosynthetic enzymes as described previously [177–180] (reviewed in [113]).

## Cystathionine-β-synthase knockout mouse models

Watanabe and colleagues were the first group to report on the generation of a CBS deficiency mouse line using gene targeting of embryonic stem (ES) cells followed by incorporation into C57BL/6J mice. This early work establishes an in vivo system to explore aspects relating to homocysteine and its associated pathophysiological effects in cardiovascular diseases. Homozygous animals completely lacked CBS and mice suffer from severe homocysteinemia, have severe growth retardation and many die within 5 weeks following birth. Heterozygous animals show greater viability and have a 50% reduction in CBS expression and enzyme activity in the liver and have twice normal plasma homocysteine levels. Studies using this model are, therefore, restricted to younger animals and may consequently be influenced by the age-dependent expression of other H_2_S biosynthetic enzymes, such as CSE. For this reason, some authorities have called into question the use of this model [177].

Problems associated with early lethality in the CBS model were later overcome by the work of Wang et al. [178, 179]. In the first approach taken by this group, mice were produced with the aim of overexpressing CBS. This was achieved using a transgenic system in which the human CBS cDNA was placed under the control of the zinc-inducible metallothionein promoter (Tg-CBS). Zinc supplementation in Tg-CBS mice causes a two–four-fold increase in liver and kidney CBS activity and a 45% decrease in serum homocysteine levels. In contrast to previous model systems, these animals do not develop hepatic steatosis, fibrosis, or suffer from high rates of neonatal death. The second approach was to engineer mice that express the human I278T and I278T/T424N mutant CBS proteins under the control of a metallothionein driven transgene. These animals were rescued from early lethality yet still showed severe elevations in both plasma and tissue levels of homocysteine, methionine, *S*-adenosylmethionine, and *S*-adenosylhomocysteine and a concomitant decrease in plasma and tissue levels of cysteine [178]. Finally, MacClean and colleagues developed a mouse model null for the mouse CBS gene that carried copies of the human CBS gene expressed at low levels [180]. So far, CBS KO models have supported a range of studies focused on folate metabolism [181, 182], blood brain barrier function [183], endothelial dysfunction [184], cerebral vascular dysfunction [185], brain function linked to changes in the SAPK/JNK signalling pathway [186], redox homeostasis [187–189] microvascular remodelling [190], blood–brain barrier integrity [191], lung fibrosis [192], lipid homeostasis [193–195], retinal neuron death [196], infertility [197, 198], and susceptibility to drug induced toxicity [199]. Of relevance here then is the growing body of work indicating that H_2_S plays a part in many of these processes.

## Cystathionine γ-lyase knockout mouse models

So far, the most widely used animal system in H_2_S research is the CSE-KO model. To date, CSE-KO animals have been utilised to explore the role of H_2_S within the cardiovascular disease [204], diabetes [200, 201, 213], and in studying interactions of H_2_S with other important gaseous signalling molecules, such as nitric oxide [202]. The production of viable and fertile CSE-KO animals was first reported in the work of Yang et al. In these homozygous animals, CSE mRNA and protein levels were absent in heart, aorta, mesenteric artery, liver, and kidneys. Importantly, both tissue and serum levels of H_2_S were significantly reduced in KO animals with this correlated with an age-dependent increase in blood pressure and impaired endothelium-dependent vasorelaxation [204]. This is in contrast to the CSE-KO model reported by Ishii et al. [203], in which animals appeared both normotensive and hyperhomocysteinemic. Interestingly, these mice were extremely sensitive to sulfur amino-acid restriction and homozygous animals maintained on a low cysteine diet, succumbed to acute skeletal muscle atrophy, and reduced tissue glutathione levels and lethality. Hepatocytes isolated from these animals were also highly sensitive to oxidative stress. To date, the CSE-KO model developed by Yang has been widely used to explore the role of H_2_S across a range of pathophysiological conditions. These studies are summarised in Table 2 and include hypertension [204], cellular proliferation [205], oxygen sensing [206], cellular senescence [207] pressure overload heart failure [208], asthma [209], vasorelaxation [210], ischemia/reperfusion injury [202, 211], atherosclerosis [212], caerulein-induced acute pancreatitis [213], postischemic cerebral vasodilation/hyperemia [214], arteriogenesis [215], pain [216], gluconeogenesis [217], M2 macrophage polarization [45], antiviral effects [218], and infiltration and migration [219]. Particularly interesting are the functional aspects relating to interaction of H_2_S with other gaseous signalling molecules. It is now widely accepted that H_2_S and NO readily interact at physiological pH to produce a range of biologically active species [65, 220–222]. An established link between NO and H_2_S has now been reported utilising the CSE-KO systems. Studies by Kondo and colleagues reported on the influence of H_2_S and its interaction with NO in a murine model of pressure overload-induced heart failure using CSE-KO animals [208]. CSE knockout (KO) animals had reduced circulating H_2_S levels and cardiac dilatation and dysfunction. In this instance, H_2_S therapy was found to be cardioprotective. This corresponding with the upregulation of the VEGF-Akt-eNOS-nitric-oxide-cGMP pathway, preserved mitochondrial function, attenuated oxidative stress, and increased myocardial vascular density. Elevated oxidative stress, dysfunctional eNOS, diminished NO levels, and exacerbated myocardial and hepatic I/R injury are also reported for CSE-KO animals [202]. Collectively, this work suggesting that H_2_S and NO interact and that H_2_S is particularly important in the regulation of NO within the cardiovascular system.

**Table 2:** Available CSE knockout mice models have been used to confirm a role of H_2_S across a wide range of pathophysiological models

Biological process	Consequence	References
Vasorelaxation and hypertension	Genetic deletion of CSE in mice markedly reduces H_2_S levels in the serum, heart, aorta, and other tissues. Mutant mice lacking CSE display pronounced hypertension and diminished endothelium-dependent vasorelaxation	[204]
Cell proliferation and apoptosis	CSE-KO mice have lower levels of phosphorylated extracellular signal-regulated kinase (ERK1/2) in mesentery arteries. SMCs of KO animals display an increased proliferation rate in vitro and in vivo, and these cells are more susceptible to apoptosis	[205]
O_2_ sensing	Deletion of CSE severely impairs carotid body response and ventilatory stimulation to hypoxia, as well as a loss of hypoxia-evoked H_2_S generation	[206]
Cellular senescence	Mouse embryonic fibroblasts isolated from CSE knockout mice (CSE-KO-MEFs) display increased oxidative stress and accelerated cellular senescence. The protein expression of p53 and p21 is significantly increased in KO-MEFs, and knockdown of p53 or p21 reversed CSE deficiency-induced senescence	[207]
Pressure overload-induced heart failure	H_2_S levels are decreased in mice following heart failure. CSE plays a critical role in the preservation of cardiac function in heart failure	[208]
Asthma	CSE expression was absent and H_2_S production rate significantly lower in the lungs of CSE-KO mice. CSE deficiency resulted in aggravated AHR, increased airway inflammation, and elevated levels of Th2 cytokines IL-5, IL-13, and eotaxin-1 in bronchoalveolar lavage fluid after OVA challenge	[209]
Physiologic vasorelaxation	CSE-KO induces elevated resting-membrane potential of SMCs and eliminated methacholine-induced endothelium-dependent relaxation of mesenteric arteries. H_2_S is an endothelium derived hyperpolarizing factor	[210]
Renal ischemia/reperfusion	CSE-KO mice have markedly reduced renal production of H_2_S, and CSE deficiency increases damage and mortality after renal ischemia/reperfusion injury as compared to wild-type mice	[211]
Atherosclerosis	Deficiency of CSE in mice leads to a decreased endogenous H_2_S levels, and age-dependent increase in blood pressure, and impaired endothelium-dependent vasorelaxation. CSE-KO animals fed with an atherogenic diet developed early fatty streak lesions in the aortic root, elevated plasma levels of cholesterol and low-density lipoprotein cholesterol, hyperhomocysteinemia, increased lesional oxidative stress and adhesion molecule expression, and enhanced aortic intimal proliferation	[212]
Caerulein-induced acute pancreatitis	CSE-KO mice showed significantly less local pancreatic damage as well as acute pancreatitis-associated lung injury compared with the WT mice. Lower levels of pancreatic eicosanoid and cytokines, as well as reduced acinar cell NF-κB activation in the CSE-KO mice	[213]
Ischemia/reperfusion (I/R) injury	CSE-KO mice exhibit elevated oxidative stress, dysfunctional eNOS, diminished NO levels, and exacerbated myocardial and hepatic I/R injury. H_2_S therapy restored eNOS function and NO bioavailability and attenuated I/R injury	[202]
Postischemic cerebral vasodilation/hyperemia	CSE-KO reduced postischemic cerebral vasodilation/hyperemia but only inhibited Na-F extravasation. Upregulated CBS was found in cerebral cortex of CSE-KO animals. L-cysteine-induced hydrogen sulfide (H_2_S) production is similarly increased in ischemic side cerebral cortex of control and CSE-KO mice	[214]
Arteriogenesis	Femoral artery ligation of WT mice significantly increased CSE activity, expression and endogenous H_2_S generation in ischaemic tissues, and monocyte infiltration. These being largely absent in CSE-KO mice. Treatment of CSE-KO mice with the polysulfide donor diallyl trisulfide restored ischaemic vascular remodelling, monocyte infiltration, and cytokine expression	[215]
Pain	Paw inflammation and peripheral nerve injury causes the upregulation of CSE expression in dorsal root ganglia. CSE-KO mice demonstrated normal pain behaviours in inflammatory and neuropathic pain models. This finding suggestive that CSE is not critically involved in chronic pain signaling in mice and that sources different from CSE mediate the pain relevant effects of H_2_S	[216]
Gluconeogenesis	CSE-KO mice reduced gluconeogenesis, which was reversed by administration of NaHS (an H_2_S donor). H_2_S upregulates the expression levels of peroxisome proliferator-activated receptor-γ coactivator-1α and phosphoenolpyruvate carboxykinase. Upregulation of PGC-1α is mediated via the GR pathway and through the activation of the cAMP/PKA pathway. PGC-1α, and the activities of glucose-6-phosphatase and fructose-1,6-bisphosphatase are increased via S-sulfhydration	[217]
Mitochondrial biogenesis-dependent M2 polarization of macrophages	H_2_S supplementation ameliorated pathological remodeling and dysfunction post-MI in WT and CSE-KO mice. Decreased infarct size and mortality, accompanied by an increase in the number of M2-polarized macrophages at the early stage of MI. H_2_S induced M2 polarization was achieved by enhanced mitochondrial biogenesis and fatty acid oxidation	[45]
Antiviral	H_2_S has antiviral and anti-inflammatory activity in respiratory syncytial virus (RSV) infection. CSE-KO mice showed significantly enhanced RSV-induced lung disease and viral replication compared to wild-type animals. Intranasal delivery of GYY4137 to RSV-infected mice significantly reduced viral replication and markedly improved clinical disease parameters and pulmonary dysfunction	[219]
Infiltration and migration	Increased infiltration of macrophages into the infarcted myocardium at early stage of MI cardiac tissues in CSE-KO mice. Treatment with the H_2_S donor NaHS enhances macrophage migration. This is achieved by accelerating internalization of integrin β1 and activating downstream Src-FAK/Pyk2-Rac pathway	[220]

Many of these studies have shown that loss of H_2_S synthesising capacity within tissues significantly affects the cardiovascular system, metabolism, and recovery from stress insults. Such studies highlight a fundamental role of H_2_S in the regulation of cellular stress pathways and in physiological responses to stress

Several newer reports have focused on the overexpression of CSE within mammalian systems. For example, in the work of Elrod et al, a transgenic mouse model was developed in which CSE is overexpressed within cardiac tissues leading to increased myocardial levels of H_2_S [37]. These mice had a reduction in infarct size following MI-R injury and were used to establish that a localised increase of H_2_S within cardiac tissues protects against myocardial infarction. Similarly, manipulation of CSE either via knockdown or overexpression in mammalian cells has also shed additional light on the cardioprotective effects of H_2_S. Wang and colleagues found that CSE overexpression reduces ox-LDL-stimulated tumor necrosis factor-α (TNF-α) generation in Raw264.7 and primary macrophage, while CSE knockdown enhanced it [149]. Under pathophysiological conditions linked to CVD, Cheung et al. reported that overexpression of CSE reduces markers associated with atherosclerosis [223]. Using transgenic ApoE knockout mice overexpressing CSE (Tg/KO), increased endogenous H_2_S production in aortic tissue was demonstrated that correlated with reduced atherosclerotic plaque sizes and reduced plasma lipid profiles in mice maintained on an atherogenic diet. Moreover, an upregulation in plasma glutathione peroxidase, indicative of reduced oxidative stress, and an increase in the expression of p-p53 and downregulation of inflammatory nuclear factor-kappa B (NF-κB) were noted [223]. Decreased CSE expression and its influence on H_2_S metabolism and atherosclerosis are currently an active area of investigation. Utilising the CSE knockout mouse, Mani et al. revealed a functional role of the CSE enzyme in atherosclerosis development [212]. In CSE-KO animals, maintained on an atherogenic diet, cholesterol levels were found to be twofold higher within the plasma of CSE-KO animals compared to the WT animals. Moreover, fatty acid streaks, atherogenic lesions, and reduced blood flow were seen in CSE-KO animals. In this instance, KO animals treated with NaHS for 12 weeks showed significant improvements in plasma lipid profiles and decreased atherosclerotic lesions thus confirming a role of H_2_S in atherosclerosis. Furthermore, by combining the CSE-KO with the ApoE-1 KO genetic background to produce a double KO system (DKO), the authors were again able to demonstrate reduced lesion formation in DKO animals when treated with NaHS [212]. Thus, endogenous loss of CSE has been shown to increase disease severity across several independent studies utilising the CSE-KO model.

## 3-Mercaptopyruvate sulfurtransferase knockout mouse models

The roles for both CBS and CSE and their part played in the production of H_2_S within biological systems have been broadly defined in recent years, yet the view that these two enzymes are perhaps the only ones responsible for maintaining physiological levels of H_2_S is rather simplistic. As mentioned, an additional enzymatic system is known, that of 3-MST [224]. In view of this, efforts have been made to generate a 3-MST murine model that could potentially provide a detailed picture of how this enzyme functions and its role in diseases [227]. From a biochemical perspective 3-MST is a multifunctional enzyme involved in (1) cysteine catabolism, since it catalyses the trans-sulfuration of the substrate 3-mercaptopyruvate to pyruvate and (2) functions in cyanide detoxification. Also, the protein has a potential redox function since in the presence of the oxidant hydrogen peroxide (H_2_O_2_), enzyme activity is inhibited [225]. Oxidant-mediated inhibition appears to occur via the formation of a sulfenate (SO^−^) moiety at the catalytic site cysteine. Enzymatic activity can be re-established in the presence of reducing agents DTT or reduced thioredoxin but not the cellular antioxidant glutathione. Under conditions of mild oxidative stress, such as those found in physiological systems, 3-MST activity is reduced leading to a resultant increase in cysteine concentrations in vitro. Thus, the current views suggest that 3-MST serves as an antioxidant protein. The curious fact that this enzyme is localised to mitochondria has further bolstered work on this enzyme, especially given the known inhibitory effects of H_2_S on cytochrome c oxidase function [226]. Ongoing work in this area has shown that 3-mercaptopyruvate stimulates mitochondrial H_2_S production that in turn stimulates electron transport and bioenergetics at low concentrations (10–100 nM). Conversely, siRNA-mediated silencing of 3-MST reduces basal bioenergetics and prevents the stimulatory effects of 3-MP on mitochondrial energetics. In this scenario, H_2_S can be seen to serve as an electron donor that functions as an inorganic source of energy that supports electron transport and ATP production in mammalian cells. Interestingly, oxidant-mediated stress reverses these effects in cells. Shibuya reported that that tissue levels and production of H_2_S within brain tissues were similar in CBS KO mice with this supporting the notion that an alternate H_2_S production system must exist within brain tissues [87]. Indeed, this work confirmed that CBS was not the primary source of H_2_S within this organ. Further characterisation led to the realisation that two proteins work in concert to produce H_2_S within brain tissues, these being, cysteine aminotransferase and 3-MST respectively [87]. While a 3-MST-KO model has been developed currently only one report exists citing the generation and utilised of this model. Nagahara et al. were the first to describe a homozygous (null) MST-knockout (MST-KO) mouse model [227]. These mice have increased anxiety-like behaviour, with increased serotonin levels in the prefrontal cortex. In this instance, 3-MST was proposed to function as an antioxidant redox-sensing protein involved in maintaining cellular redox homeostasis.

## Genetic models of H_2_S detoxification systems in animals

Three major enzymatic routes for the removal of H_2_S from tissues are currently recognised these constituting the aforementioned proteins SQR, ETHE1, and CDO. At present, the sites and rates of H_2_S detoxification have been less well defined than that of the biosynthetic routes of production. However, these systems likely play an equally important function in maintaining physiologically relevant tissue concentrations of H_2_S. Changes in the expression levels of these proteins would alter the physiological concentrations of this sulfurous gas in vivo and therefore, the response of cells to exposure to this molecule. Even with their recognised association with H_2_S detoxification, only now are we beginning to see how these enzymes influence physiological levels of this gas.

## Sulfide–quinone reductase-like protein knockout models

In mammalian systems, sulfide is oxidized by the mitochondrial sulfide–quinone reductase-like protein (SQR), a homologue of bacterial sulfide–ubiquinone oxidoreductase (SQR), and fission yeast heavy metal tolerance 2 protein [228]. This protein is involved in the transfer of an electron from sulfide to membrane intrinsic quinones [229]. The process of sulfide oxidation, therefore, links sulfide catabolism to oxidative phosphorylation and the subsequent production of ATP. This whole process allowing for sulfide to be used as an inorganic substrate for the human electron transfer chain. SQR is a component of several mammalian tissues, and protein expression has been confirmed within heart, lung, colon, liver, kidney, thyroid, brain, leukocytes, and penis and testicles of mice and rats [230]. Fractionation experiments revealed this protein to be localised to mitochondria. SQR mRNA levels can be increased following exposure to sulfide in T cells and also with increasing age within the kidney. This finding indicating that the expression levels of this protein show some plasticity that allows for SQR to respond to changes in tissue H_2_S levels. It is easy to envisage that changes in SQR protein levels would influence H_2_S oxidation rates and the role of H_2_S in the production of ATP, ROS formation, oxygen sensing [231] and subsequently the effects of this gas on cell-signalling networks [1] and on S-sulfhydration of proteins [62]. Recently, polymorphisms have been identified for the SQR gene, which are linked to pathophysiological conditions in humans. Jin et al. reported on the SQR I264T gene variant that increases susceptibility to osteoporosis in Korean postmenopausal women [232]. In another study, genomic-wide screening in Filipino women reported that the rs12594514 SNP in the SQR gene is associated with two obesity-related phenotypes [233]. Interestingly, the cellular levels of H_2_S are critical determinants in the regulation of bone remodelling [169, 232] and osteoclast differentiation [234, 235]. Moreover, it is now widely recognised that H_2_S has a range of functions linked to metabolism and obesity [7, 236–240]. Therefore, it is likely that SQR has the potential to influence some of the biological effects of H_2_S in vivo. To date, there are no reported murine SQR KO models however, SQR KO *C.elegans *systems are known. Using gene knockout strategies in *C. elegans*, SQR was found to be important in the maintenance of protein translation. In SQR mutant worms, exposure to H_2_S leads to phosphorylation of eIF2α and the inhibition of protein synthesis. The authors speculating that SQR may be involved in H_2_S signalling relating to proteostasis [241]. Of relevance, here is the potential link with H_2_S, proteostasis and the anti-ageing effects of this gas.

## Ethylmalonic encephalopathy knockout mouse models

Another candidate protein potentially involved in H_2_S detoxification is that of ETHE1. The ETHE1 gene codes for an iron-containing protein from the metallo β-lactamase family are required in the mitochondrial sulfide oxidation pathway and for the oxidation of glutathione persulfide (GSSH) to give glutathione and persulfate [91]. ETHE1 protein catalyses the second step in the mitochondrial sulfide oxidation pathway downstream of SQR. Mutations in this gene cause the rare condition known as ethylmalonic encephalopathy (EE) that affects the brain, gastrointestinal tract, and peripheral vessels [242]. This inborn error of metabolism is an autosomal recessive condition that is invariably fatal and characterised by encephalopathy, microangiopathy, chronic diarrhea, and defective cytochrome c oxidase (COX) in muscle and brain [243]. The latter oxidizes H_2_S to persulfide and transfers electrons to the electron transport chain via reduced quinone. Indeed, recombinant expression of human SQR is known to enhance sulfide oxidation in mammalian cells [244]. More revealing insights as to the functional role of ETHE1 have been reported [245]. Adopting a proteomic approach Hildbrant and colleagues conducted an analysis of ETHE1 KO mouse tissues and confirmed a role of ETHE1 in the sulfide oxidation pathway while also revealing more subtle effects on post-translational protein modifications linked to protein cysteine modification. Elevated H_2_S levels caused by loss of ETHE1 likely cause an increase in S-sulfhydration of cellular proteins via persulfide-mediated reactions [246]. Of particular interest, from this work is that sulfide signalling seems to play a pivotal part in regulating mitochondrial catabolism of fatty acids and branched-chain amino acids. Interestingly, sulfide concentrations are decreased in the plasma of overweight men and low sulfide levels are associated with the development of insulin resistance in Type 2 diabetes [247]. Moreover, in rats fed high-fat diets ETHE1 and SQR are reported to be decreased by more than 50% in tissues [248].

## Cysteine dioxygenase knockout mouse models

Finally, a common component linking all of the enzymatic systems described herein is their reliance on intermediates derived from sulfur amino-acid metabolism, specifically, the interplay between cysteine synthesis, its cellular uses, and its degradation. Cysteine homeostasis and the relative rates of synthesis versus degradation will clearly influence how and when H_2_S will be produced within tissues. This coupled with the relative rates of oxidation of both molecules further adding complexity to the H_2_S story. One particularly interesting model is the cysteine dioxygenase (CDO; EC: 1.13.11.20) KO mouse model. Ordinarily, CDO oxidizes cysteine-to-cysteine sulfinate, which is further metabolized to either taurine or to pyruvate plus sulfate. This metabolic pathway is believed to function in maintaining cysteine levels and to supply circulatory taurine. In the CDO KO mouse line, there is postnatal mortality, growth deficit, and connective tissue pathology. Moreover, KO animals have reduced taurine levels, elevated cysteine levels, and increased desulfuration in liver tissues that correlates with the elevated production of H_2_S. This reported to be due to CBS activation. Importantly, CDO null mice also exhibit lower hepatic cytochrome c oxidase levels, suggesting impaired electron transport capacity. Cytochrome c oxidase being a known cellular target prone to H_2_S-mediated inhibition. Similarly, in hepatocytes isolated from CDO null mice increased synthesis of H_2_S within cells occurs that is perhaps due to an increase in the endogenous pool of cysteine within tissues [249]. Also reported in the CDO KO mice is an increase in the urinary excretion of thiosulfate, coupled with higher tissue and serum cystathionine and lanthionine levels. Importantly, the inhibition and destabilization of cytochrome c oxidase are observed that again is consistent with increased production of H_2_S [249, 250]. Thus, it would appear that the ability of CDO to control cysteine levels may be necessary to maintain low H_2_S/sulfane sulfur pools within tissues to facilitate the use of H_2_S as a signalling molecule [251]. This model, therefore, provides a unique system to explore cysteine metabolism and its influence of H_2_S production and redox-signalling networks.

## Availability of knockout mouse models for H_2_S research

At this time, it may be of interest to researchers that CBS KO mice are now commercially available and can be obtained from the Jackson laboratories which supplies the JAX^®^ Mice derived from the fully sequenced mouse strain, C57BL/6J [252]. This particular line is useful for studying the in vivo role of elevated levels of homocysteine in the aetiology of cardiovascular diseases and was developed in the lab of Dr Nobuyo Maeda at the University of North Carolina at Chapel Hill. A number of researchers have utilised this mouse model to determine the functional role of H_2_S in colitis [253] for the role of H_2_S in alveolarization [254] and in the prevention of hyperhomocysteinemia associated chronic renal failure [255], however, studies are limited primarily due to the high mortality rates in offspring. In the case of research using CSE knockout (CSE-KO) animals, this model is more widely reported in the literature. These animals have markedly reduced H_2_S levels in the serum, heart, aorta, and other tissues and mutant mice lacking CSE display pronounced hypertension and diminished endothelium-dependent vasorelaxation. Again, this model is particularly useful for studying cardiovascular disease. Although not commercially available at present several institutions maintain the CSE-KO mouse model that was originally developed in the laboratory of Rui Wang, Lakehead University, Thunder Bay, Ontario, Canada. This model is the most widely used physiologically relevant model and has been the focus of research ranging from the role of H_2_S in vasorelaxation [204], to O_2_ sensing in the carotid body [206]. 3-MST and ETHE1 KO animals are maintained at the Isotope Research Centre, Nippon Medical School, Tokyo and at the Institute of Neurology Carlo Besta-Istituto di Ricovero e Cura a Carattere Scientifico Foundation, Milan, Italy. Hopefully in the future, these models will become more common place in research focused on H_2_S biology.

## Non-mammalian genetic models

The majority of work highlighting a biological role for H_2_S has been derived from mammalian models. Information derived from non-mammalian models reflects on the evolutionary importance of H_2_S and its role in biochemical and physiological processes across different taxa. Several reports now describe the homeostatic systems and physiological effects of H_2_S across a range of animal and plant systems particularly in the model organisms *C. elegans*, *D. melanogaster*, *D. rerio,* and *Arabidopsis thaliana* [256, 257]. The reason for this work is one of translation, since, for example, the exploitation of the H_2_S biosynthetic pathway in animals and in plants may assist in Agritech for the purpose of improving crop yields or resistance to pathogen attack. To date, only a handful of studies have been described in which the targeted deletion or overexpression of H_2_S synthesising enzymes has been manipulated in non-mammalian systems. Much of this work has utilised molecular approaches to alter the expression levels of H_2_S synthesising enzymes in the nematode worm, *C. elegans.* These studies have identified roles for H_2_S in the ageing process, in longevity, and in the health benefits attributed to caloric/dietary restriction. It is widely known that worms exposed to exogenous H_2_S have increased longevity and thermotolerance [258, 259]. However, direct molecular confirmations that these physiological processes can be controlled via endogenous H_2_S synthesis have only recently been described [54, 55]. In these studies, siRNA-mediated silencing approaches were utilised to knock down *C. elegans* targets. Deletion of CYST-2, a cysteine sulfhydrylase, caused a significant reduction in lifespan in worms exposed to stress conditions [54]. This finding establishing a clear link between H_2_S synthesis and the ability of worms to adapt and recover from stress insults associated with the ageing process. Indeed, deficiency in mpst-1, mammalian 3-MST orthologue 1, reduces lifespan in *C. elegans*. It has subsequently been demonstrated in the work of Hine et al. that H_2_S production in *C. elegans* is linked to the health benefits attributed to caloric/dietary restriction. In this study, utilising siRNA technologies, individual KO experiments were performed that focused on a number of proteins associated with the trans-sulfuration pathway, namely, the cystathione-γ-lyase worm homologues CTH-1 and CTH-2 and the CBS homolog CBS-1 and CBL-1 [260, 261]. Loss of functional CBL-1 and CBS-2 protein appears to have no effects on longevity when expressed in the *eat*-*2* mutant worms; the *eat*-*2* mutant serving as a genetic model of life extension that mimics dietary restriction. Interestingly, *eat*-*2* worms produce more H_2_S than their wild-type counterparts. Importantly, the overexpression of CBS-1 extends the median lifespan of wild-type worms this clearly showing that H_2_S mediates the beneficial effects attributed to dietary/caloric restriction in *C. elegans*.

Similar finding has also been reported for *Drosophila melanogaster.* In this model, dietary restriction promotes the upregulation and increased activity of the trans-sulfuration pathway leading to increased tissue synthesis rates of H_2_S [262]. Transgene-mediated increases in gene expression and enzyme activity of *Drosophila* cystathionine β-synthase (dCBS) are sufficient to increase fly lifespan. Moreover, the inhibition of the trans-sulfuration pathway effectively blocks the lifespan extension normally observed in diet-restricted animals. These findings are of particular interest, since they provide an additional evidence that H_2_S plays important functional roles in the ageing process of living organisms. Besides, ageing, H_2_S also appears to mediate neurodegenerative processes in *Drosophila* models. For example, overexpression of CSE in *Drosophila* suppresses spinocerebellar ataxia type 3-associated damage and neurodegeneration [263]. The observed decreased in cellular damage being attributed to a reduction in oxidative stress and a reduced immune response in flies. Clearly, these findings correlate well with the known antioxidant and anti-inflammatory effects attributed to H_2_S.

Work using teleost’s species, such as *Danio,* are rare, but, nonetheless, provides important information on the physiological role of H_2_S. In the work of Kumai et al., H_2_S was found to influence Na^+^ homeostatic regulation in the larva of *D. rerio* [264]. Translational gene knockdown was used to reduce CSE expression in tissues. Using this approach Kumai and colleagues were able to elegantly demonstrate that H_2_S is an endogenous inhibitor of Na^+^ uptake in developing zebrafish.

## Conclusions

Over the last decade, considerable evidence has been accumulated which collectively points to a functional role for H_2_S in a number of physiological systems. Much of these data have been derived from pharmacological intervention in which inhibition of enzymatic systems linked to the production of H_2_S has been targeted or via direct drug targeting using small molecular weight H_2_S donor molecules. Invariably, these studies have highlighted a role of H_2_S levels within a number of pathophysiological states and that restoration of tissue H_2_S levels is protective in the majority of cases. Despite the current knowledge, and continued breakthroughs, one can envisage that transgenic models will be at the forefront of future work in this area. Developments based on the approach taken by Mani et al. in which a double knockout mouse model in which both the CSE and the apolipoprotein E gene are silenced may be particularly revealing [212]. Studies using these models have been fruitful and have shown how changes in cellular H_2_S levels influence physiological processes. Yet, the true power of these models is still to be realised. Since the discovery that cross talk exists between H_2_S with other gaseous signalling molecules, such as NO, the use of transgenic models in which one or both sets of synthesising enzymes are silenced may be invaluable in future studies. Data on the interactions of NO with H_2_S are only just emerging and it would be fascinating to explore the effects of incorporating the CSE-KO background into other transgenic systems such as that of iNOS [265] or eNOS KO [266, 267] mouse models. How would the loss of each gas alter the formation and levels of circulatory nitrosothiols for example? What would be the consequences of this systemically? Could biologically active persulfides compensate for the loss of nitrosothiols? More revealing is the current evidence showing that both gases can influence mitochondrial function, energy metabolism, and tissue homeostasis, but the functional consequences of combined defects in H_2_S and NO production are not known. Could these interactions, or lack off, underpin dysregulation in metabolism as seen in diabetes or obesity? The development of these models would also be particularly useful in the screening of H_2_S/NO hybrid donor drugs [268–270]. Finally, could double knockout models be developed to explore the influence of H_2_S detoxification enzymes on cardiovascular function and on inflammatory responses in animals? What, for example, would be the effect of loss of CBS, or 3-MST in the apolipoprotein E KO murine model? Would this further predisposes animals to atherosclerosis, and would similar effects be found with the overexpression of SQR and ETHE1? With the development of these transgenic models, there are certainly more questions than answers and much remains to be explored regarding the role of this gas within biological systems. Hopefully, a greater understanding will come from the use of these newer tools that will hopefully assist in the development and introduction of new H_2_S releasing pro-drugs within the clinic.

## References

[1] Li L, Rose P, Moore PK (2011). Hydrogen sulfide and cell signaling. Annu Rev Pharmacol Toxicol.

[2] Kabil O, Vitvitsky V, Banerjee R (2014). Sulfur as a signaling nutrient through hydrogen sulfide. Annu Rev Nutr.

[3] Gemici B, Elsheikh W, Feitosa KB (2015). H_2_S-releasing drugs: anti-inflammatory, cytoprotective and chemopreventative potential. Nitric Oxide.

[4] Yang G, Sun X, Wang R (2004). Hydrogen sulfide-induced apoptosis of human aorta smooth muscle cells via the activation of mitogen-activated protein kinases and caspase-3. FASEB J.

[5] Shi S, Li QS, Li H (2009). Anti-apoptotic action of hydrogen sulfide is associated with early JNK inhibition. Cell Biol Int.

[6] Hu Y, Chen X, Pan TT (2008). Cardioprotection induced by hydrogen sulfide preconditioning involves activation of ERK and PI3K/Akt pathways. Pflugers Arch.

[7] Manna P, Jain SK (2011). Hydrogen sulfide and l-cysteine increase phosphatidylinositol 3,4,5-trisphosphate (PIP3) and glucose utilization by inhibiting phosphatase and tensin homolog (PTEN) protein and activating phosphoinositide 3-kinase (PI3K)/serine/threonine protein kinase (AKT)/protein kinase Cζ/λ (PKCζ/λ) in 3T3l1 adipocytes. J Biol Chem.

[8] Pan TT, Neo KL, Hu LF (2008). H_2_S preconditioning-induced PKC activation regulates intracellular calcium handling in rat cardiomyocytes. Am J Physiol Cell Physiol.

[9] Szabo G, Veres G, Radovits T (2011). Cardioprotective effects of hydrogen sulphide. Nitric Oxide.

[10] Calenic B, Yaegaki K, Ishkitiev N (2013). p53-Pathway activity and apoptosis in hydrogen sulfide-exposed stem cells separated from human gingival epithelium. J Periodontal Res.

[11] Lee HG, Mariappan MM, Feliers D (2012). Hydrogen sulfide inhibits high glucose-induced matrix protein synthesis by activating AMP-activated protein kinase in renal epithelial cells. J Biol Chem.

[12] Cai J, Shi X, Wang H (2016). Cystathionine γ lyase-hydrogen sulfide increases peroxisome proliferator-activated receptor γ activity by sulfhydration at C139 site thereby promoting glucose uptake and lipid storage in adipocytes. Biochim Biophys Acta.

[13] Li X, Zhang KY, Zhang P (2014). Hydrogen sulfide inhibits formaldehyde-induced endoplasmic reticulum stress in PC12 cells by upregulation of SIRT-1. PLoS One.

[14] Xei L, Feng H, Li S (2016). SIRT3 mediates the antioxidant effect of hydrogen sulfide in endothelial cells. Antioxid Redox Signal.

[15] Talaei F, van Praag VM, Henning RH (2013). Hydrogen sulfide restores a normal morphological phenotype in Werner syndrome fibroblasts, attenuates oxidative damage and modulates mTOR pathway. Pharmacol Res.

[16] Zayachkivska O, Havryluk O, Hrycevych N (2014). Cytoprotective effects of hydrogen sulfide in novel rat models of non-erosive esophagitis. PLoS One.

[17] Meng JL, Mei WY, Dong YF (2011). Heat shock protein 90 mediates cytoprotection by H_2_S against chemical hypoxia-induced injury in PC12 cells. Clin Exp Pharmacol Physiol.

[18] Yang C, Yang Z, Zhang M (2011). Hydrogen sulfide protects against chemical hypoxia-induced cytotoxicity and inflammation in HaCaT cells through inhibition of ROS/NF-κB/COX-2 pathway. PLoS One.

[19] Yang M, Huang Y, Chen J (2014). Activation of AMPK participates hydrogen sulfide-induced cyto-protective effect against dexamethasone in osteoblastic MC3T3-E1 cells. Biochem Biophys Res Commun.

[20] Calvert JW, Jha S, Gundewar S (2009). Hydrogen sulfide mediates cardioprotection through Nrf2 signaling. Circ Res.

[21] Li L, Bhatia M, Zhu YZ (2005). Hydrogen sulfide is a novel mediator of lipopolysaccharide-induced inflammation in the mouse. FASEB J.

[22] Zanardo RC, Brancaleone V, Distrutti E (2006). Hydrogen sulfide is an endogenous modulator of leukocyte-mediated inflammation. FASEB J.

[23] Whiteman M, Li L, Rose P (2010). The effect of hydrogen sulfide donors on lipopolysaccharide-induced formation of inflammatory mediators in macrophages. Antioxid Redox Signal.

[24] Gemici B, Wallace JL (2015). Anti-inflammatory and cytoprotective properties of hydrogen sulfide. Methods Enzymol.

[25] Zhao W, Zhang J, Lu Y (2001). The vasorelaxant effect of H(2)S as a novel endogenous gaseous K(ATP) channel opener. EMBO J.

[26] Köhn C, Schleifenbaum J, Szijártó IA (2012). Differential effects of cystathionine-γ-lyase-dependent vasodilatory H_2_S in periadventitial vasoregulation of rat and mouse aortas. PLoS One.

[27] Yang R, Teng X, Li H (2016). Hydrogen sulfide improves vascular calcification in rats by inhibiting endoplasmic reticulum stress. Oxid Med Cell Longev.

[28] Wang Z, Liu D-X, Wang F-W (2013). l-Cysteine promotes the proliferation and differentiation of neural stem cells via the CBS/H_2_S pathway. Neuroscience.

[29] Cai WJ, Wang MJ, Moore PK (2007). The novel proangiogenic effect of hydrogen sulfide is dependent on Akt phosphorylation. Cardiovasc Res.

[30] Wallace JL, Dicay M, McKnight W (2007). Hydrogen sulfide enhances ulcer healing in rats. FASEB J.

[31] Papapetropoulos A, Pyriochou A, Altaany Z (2009). Hydrogen sulfide is an endogenous stimulator of angiogenesis. Proc Natl Acad Sci USA.

[32] Liu W, Liu K, Ma C (2014). Protective effect of hydrogen sulfide on hyperbaric hyperoxia-induced lung injury in a rat model. Undersea Hyperb Med.

[33] Wang G, Li W, Chen Q (2015). Hydrogen sulfide accelerates wound healing in diabetic rats. Int J Clin Exp Pathol.

[34] Jang H, Oh MY, Kim YJ (2014). Hydrogen sulfide treatment induces angiogenesis after cerebral ischemia. J Neurosci Res.

[35] Yang GD, Wang R (2007). H(2)S and cellular proliferation and apoptosis. Sheng Li Xue Bao.

[36] Baskar R, Bian J (2011). Hydrogen sulfide gas has cell growth regulatory role. Eur J Pharmacol.

[37] Elrod JW, Calvert JW, Morrison J (2007). Hydrogen sulfide attenuates myocardial ischemia-reperfusion injury by preservation of mitochondrial function. Proc Natl Acad Sci USA.

[38] Goubern M, Andriamihaja M, Nübel T (2007). Sulfide, the first inorganic substrate for human cells. FASEB J.

[39] Módis K, Coletta C, Erdélyi K (2013). Intramitochondrial hydrogen sulfide production by 3-mercaptopyruvate sulfurtransferase maintains mitochondrial electron flow and supports cellular bioenergetics. FASEB J.

[40] Modis K, Asimakopoulou A, Coletta C (2013). Oxidative stress suppresses the cellular bioenergetic effect of the 3-mercaptopyruvate sulfurtransferase/hydrogen sulfide pathway. Biochem Biophys Res Commun.

[41] Guo Z, Li CS, Wang CM (2015). CSE/H2S system protects mesenchymal stem cells from hypoxia and serum deprivation-induced apoptosis via mitochondrial injury, endoplasmic reticulum stress and PI3K/Akt activation pathways. Mol Med Rep.

[42] Banu S, Ravindran S, Kurian GA (2016). Hydrogen sulfide post-conditioning preserves interfibrillar mitochondria of rat heart during ischemia reperfusion injury. Cell Stress Chaperones.

[43] Szczesny B, Módis K, Yanagi K (2014). AP39, a novel mitochondria-targeted hydrogen sulfide donor, stimulates cellular bioenergetics, exerts cytoprotective effects and protects against the loss of mitochondrial DNA integrity in oxidatively stressed endothelial cells in vitro. Nitric Oxide.

[44] Coletta C, Módis K, Szczesny B (2015). Regulation of vascular tone, angiogenesis and cellular bioenergetics by the 3-mercaptopyruvate sulfurtransferase/H_2_S pathway: functional impairment by hyperglycemia and restoration by dl-α-lipoic acid. Mol Med.

[45] Miao L, Shen X, Whiteman M (2016). Hydrogen sulfide mitigates myocardial infarction via promotion of mitochondrial biogenesis-dependent M2 polarization of macrophages. Antioxid Redox Signal.

[46] Sun A, Wang Y, Liu J (2016). Exogenous H_2_S modulates mitochondrial fusion-fission to inhibit vascular smooth muscle cell proliferation in a hyperglycemic state. Cell Biosci.

[47] Zhao FL, Fang F, Qiao PF (2016). AP39, a mitochondria-targeted hydrogen sulfide donor, supports cellular bioenergetics and protects against Alzheimer’s disease by preserving mitochondrial function in APP/PS1 mice and neurons. Oxid Med Cell Longev.

[48] Vicente JB, Malagrinò F, Arese M (2016). Bioenergetic relevance of hydrogen sulfide and the interplay between gasotransmitters at human cystathionine β-synthase. Biochim Biophys Acta.

[49] Whiteman M, Gooding KM, Whatmore JL (2010). Adiposity is a major determinant of plasma levels of the novel vasodilator hydrogen sulphide. Diabetologia.

[50] Geng B, Cai B, Liao F (2013). Increase or decrease hydrogen sulfide exert opposite lipolysis, but reduce global insulin resistance in high fatty diet induced obese mice. PLoS One.

[51] Velmurugan GV, Huang H, Sun H (2015). Depletion of H_2_S during obesity enhances store-operated Ca^2+^ entry in adipose tissue macrophages to increase cytokine production. Sci Signal.

[52] Candela J, Velmurugan GV, White C (2016). Hydrogen sulfide depletion contributes to microvascular remodeling in obesity. Am J Physiol Heart Circ Physiol.

[53] Jamroz-Wiśniewska A, Gertler A, Solomon G (2015). Leptin-induced endothelium-dependent vasorelaxation of peripheral arteries in lean and obese rats: role of nitric oxide and hydrogen sulfide. PLoS One.

[54] Qabazard B, Li L, Gruber J (2013). Hydrogen sulfide is an endogenous regulator of aging in *Caenorhabditis elegans*. Antioxid Redox Signal.

[55] Qabazard B, Ahmed S, Li L (2014). *C. elegans* aging is modulated by hydrogen sulfide and the sulfhydrylase/cysteine synthase cysl-2. PLoS One.

[56] Krejcova T, Smelcova M, Petr J (2015). Hydrogen sulfide donor protects porcine oocytes against aging and improves the developmental potential of aged porcine oocytes. PLoS One.

[57] Yang G, An SS, Ji Y (2015). Hydrogen sulfide signaling in oxidative stress and aging development. Oxid Med Cell Longev.

[58] Jin S, Pu SX, Hou CL (2015). Cardiac H_2_S generation is reduced in ageing diabetic mice. Oxid Med Cell Longev.

[59] Li L, Li M, Li Y (2016). Exogenous H_2_S contributes to recovery of ischemic post-conditioning-induced cardioprotection by decrease of ROS level via down-regulation of NF-κB and JAK2-STAT3 pathways in the aging cardiomyocytes. Cell Biosci.

[60] Wei Y, Kenyon C (2016). Roles for ROS and hydrogen sulfide in the longevity response to germline loss in *Caenorhabditis elegans*. Proc Natl Acad Sci USA.

[61] Whiteman M, Armstrong JS, Chu SH (2004). The novel neuromodulator hydrogen sulfide: an endogenous peroxynitrite ‘scavenger’?. J Neurochem.

[62] Whiteman M, Cheung NS, Zhu YZ (2005). Hydrogen sulphide: a novel inhibitor of hypochlorous acid-mediated oxidative damage in the brain?. Biochem Biophys Res Commun.

[63] Mustafa AK, Gadalla MM, Sen N (2009). H_2_S signals through protein S-sulfhydration. Sci Signal.

[64] Filipovic MR (2015). Persulfidation (S-sulfhydration) and H_2_S. Handb Exp Pharmacol.

[65] Whiteman M, Li L, Kostetski I (2006). Evidence for the formation of a novel nitrosothiol from the gaseous mediators nitric oxide and hydrogen sulphide. Biochem Biophys Res Commun.

[66] Pryor WA, Houk KN, Foote CS (2006). Free radical biology and medicine: it’s a gas, man. Am J Physiol Regul Integr Comp Physiol.

[67] Cortese-Krott MM, Kuhnle GG, Dyson A (2015). Key bioactive reaction products of the NO/H_2_S interaction are S/N-hybrid species, polysulfides, and nitroxyl. Proc Natl Acad Sci USA.

[68] Olson KR (2011). A practical look at the chemistry and biology of hydrogen sulfide. Antioxid Redox Signal.

[69] Shen X, Pattillo CB, Pardue S (2011). Measurement of plasma hydrogen sulfide in vivo and in vitro. Free Radic Biol Med.

[70] Shen X, Kolluru GK, Yuan S (2015). Measurement of H_2_S in vivo and in vitro by the monobromobimane method. Methods Enzymol.

[71] Mueller EG (2014). Trafficking in persulfides: delivering sulfur in biosynthetic pathways. Nat Chem Biol.

[72] Greiner R, Pálinkás Z, Bäsell K (2013). Polysulfides link H_2_S to protein thiol oxidation. Antioxid Redox Signal.

[73] Pimentel M, Mathur R, Chang C (2013). Gas and the microbiome. Curr Gastroenterol Rep.

[74] Ida T, Sawa T, Ihara H (2014). Reactive cysteine persulfides and S-polythiolation regulate oxidative stress and redox signaling. Proc Natl Acad Sci USA.

[75] Benavides GA, Squadrito GL, Mills RW (2007). Hydrogen sulfide mediates the vasoactivity of garlic. Proc Natl Acad Sci USA.

[76] Pei Y, Wu B, Cao Q (2011). Hydrogen sulfide mediates the anti-survival effect of sulforaphane on human prostate cancer cells. Toxicol Appl Pharmacol.

[77] Tocmo R, Liang D, Lin Y (2015). Chemical and biochemical mechanisms underlying the cardioprotective roles of dietary organopolysulfides. Front Nutr.

[78] Tocmo R, Lin Y, Huang D (2014). Effect of processing conditions on the organosulfides of shallot (*Allium cepa* L. Aggregatum group). J Agric Food Chem.

[79] Liang D, Wanga C, Tocmo R (2015). Hydrogen sulphide (H_2_S) releasing capacity of essential oils isolated from organosulphur rich fruits and vegetables. J Funct Foods.

[80] Kabil O, Banerjee R (2014). Enzymology of H_2_S biogenesis, decay and signaling. Antioxid Redox Signal.

[81] Huang CW, Moore PK (2016). H_2_S synthesizing enzymes: biochemistry and molecular aspects. Handb Exp Pharmacol.

[82] Kabil O, Banerjee R (2010). Redox biochemistry of hydrogen sulfide. J Biol Chem.

[83] Xie ZZ, Liu Y, Bian JS (2016). Hydrogen sulfide and cellular redox homeostasis. Oxid Med Cell Longev.

[84] Paul BD, Snyder SH (2015). H_2_S: a novel gasotransmitter that signals by sulfhydration. Trends Biochem Sci.

[85] Saha S, Chakraborty PK, Xiong X (2016). Cystathionine β-synthase regulates endothelial function via protein S-sulfhydration. FASEB J.

[86] Lechuga TJ, Zhang HH, Sheibani L (2015). Estrogen replacement therapy in ovariectomized nonpregnant ewes stimulates uterine artery hydrogen sulfide biosynthesis by selectively up-regulating cystathionine β-synthase expression. Endocrinology.

[87] Shibuya N, Tanaka M, Yoshida M (2009). 3-Mercaptopyruvate sulfurtransferase produces hydrogen sulfide and bound sulfane sulfur in the brain. Antioxid Redox Signal.

[88] Tiranti V, Viscomi C, Hildebrandt T (2009). Loss of ETHE1, a mitochondrial dioxygenase, causes fatal sulfide toxicity in ethylmalonic encephalopathy. Nat Med.

[89] Jackson MR, Melideo SL, Jorns MS (2012). Human sulfide:quinone oxidoreductase catalyzes the first step in hydrogen sulfide metabolism and produces a sulfane sulfur metabolite. Biochemistry.

[90] Di Meo I, Fagiolari G, Prelle A (2011). Chronic exposure to sulfide causes accelerated degradation of cytochrome c oxidase in ethylmalonic encephalopathy. Antioxid Redox Signal.

[91] Kabil O, Banerjee R (2012). Characterization of patient mutations in human persulfide dioxygenase (ETHE1) involved in H_2_S catabolism. J Biol Chem.

[92] Nagahara N, Okazaki T, Nishino T (1995). Cytosolic mercaptopyruvate sulfurtransferase is evolutionarily related to mitochondrial rhodanese. J Biol Chem.

[93] Picton R, Eggo MC, Merrill GA (2002). Mucosal protection against sulphide: importance of the enzyme rhodanese. Gut.

[94] Wilson K, Mudra M, Furne J (2008). Differentiation of the roles of sulfide oxidase and rhodanese in the detoxification of sulfide by the colonic mucosa. Dig Dis Sci.

[95] Ramasamy S, Singh S, Taniere P (2006). Sulfide-detoxifying enzymes in the human colon are decreased in cancer and upregulated in differentiation. Am J Physiol Gastrointest Liver Physiol.

[96] Hirata I, Naito Y, Takagi T (2011). Endogenous hydrogen sulfide is an anti-inflammatory molecule in dextran sodium sulfate-induced colitis in mice. Dig Dis Sci.

[97] Gao Y, Yao X, Zhang Y (2011). The protective role of hydrogen sulfide in myocardial ischemia-reperfusion-induced injury in diabetic rats. Int J Cardiol.

[98] Takahashi T, Aoki Y, Okubo K (2010). Upregulation of Ca(v)3.2 T-type calcium channels targeted by endogenous hydrogen sulfide contributes to maintenance of neuropathic pain. Pain.

[99] Abeles RH, Walsh CT (1973). Acetylenic enzyme inactivators. Inactivation of gamma-cystathionase, in vitro and in vivo, by propargylglycine. J Am Chem Soc.

[100] Washtien W, Abeles RH (1977). Mechanism of inactivation of gamma-cystathionase by the acetylenic substrate analogue propargylglycine. Biochemistry.

[101] Asimakopoulou A, Panopoulos P, Chasapis CT (2013). Selectivity of commonly used pharmacological inhibitors for cystathionine β synthase (CBS) and cystathionine γ lyase (CSE). Br J Pharmacol.

[102] Steegborn C, Clausen T, Sondermann P (1999). Kinetics and inhibition of recombinant human cystathionine gamma-lyase. Toward the rational control of transsulfuration. J Biol Chem.

[103] Yao K (1975). Effects of several unusual sulfur-containing amino acids on rat liver cystathionine-gamma-lyase. Physiol Chem Phys.

[104] Thorson MK, Majtan T, Kraus JP (2013). Identification of cystathionine β-synthase inhibitors using a hydrogen sulfide selective probe. Angew Chem Int Ed Engl.

[105] Thorson MK, Van Wagoner RM, Harper MK (2015). Marine natural products as inhibitors of cystathionine beta-synthase activity. Bioorg Med Chem Lett.

[106] Zhou Y, Yu J, Lei X (2013). High-throughput tandem-microwell assay identifies inhibitors of the hydrogen sulfide signaling pathway. Chem Commun (Camb).

[107] Wing DA (1992). Modifiers of mercaptopyruvate sulfurtransferase catalyzed conversion of cyanide to thiocyanate in vitro. J Biochem Toxicol.

[108] Porter DW, Baskin SI (1995). Specificity studies of 3-Mercaptopyruvate sulfurtransferase. J Biochem Toxicol.

[109] Porter DW, Baskin SI (1996). The effect of three alpha-keto acids on 3-mercaptopyruvate sulfurtransferase activity. J Biochem Toxicol.

[110] Brosnan JT, Brosnan ME (2006). The sulfur-containing amino acids: an overview. J Nutr.

[111] Stipanuk MH (1986). Metabolism of sulfur-containing amino acids. Annu Rev Nutr.

[112] Stipanuk MH (2004). Sulfur amino acid metabolism: pathways for production and removal of homocysteine and cysteine. Annu Rev Nutr.

[113] Beard RS, Bearden SE (2011). Vascular complications of cystathionine β-synthase deficiency: future directions for homocysteine-to-hydrogen sulfide research. Am J Physiol Heart Circ Physiol.

[114] Wang J, Hegele RA (2003). Genomic basis of cystathioninuria (MIM 219500) revealed by multiple mutations in cystathionine gamma-lyase (CTH) Hum. Genet.

[115] Meier M, Oliveriusova J, Kraus JP (2003). Structural insights into mutations of cystathionine beta-synthase. Biochim Biophys Acta.

[116] Finkelstein JD (2006). Inborn errors of sulfur-containing amino acid metabolism. J Nutr.

[117] Tsai MY, Hanson NQ, Bignell M (1996). Simultaneous detection and screening of T833C and G919A mutations of the cystathionine beta-synthase gene by single-strand conformational polymorphism. Clin Biochem.

[118] Shi H, Yang S, Liu Y (2015). Study on environmental causes and SNPs of MTHFR, MS and CBS genes related to congenital heart disease. PLoS One.

[119] Ding R, Lin S, Chen D (2012). The association of cystathionine β synthase (CBS) T833C polymorphism and the risk of stroke: a meta-analysis. J Neurol Sci.

[120] Gallegos-Arreola MP, Figuera-Villanueva LE, Ramos-Silva A (2014). The association between the 844ins68 polymorphism in the CBS gene and breast cancer. Arch Med Sci.

[121] Konrad C, Müller GA, Langer C (2004). Plasma homocysteine, MTHFR C677T, CBS 844ins68 bp, and MTHFD1 G1958A polymorphisms in spontaneous cervical artery dissections. J Neurol.

[122] Zhang Z, Dai C (2002). Correlation analysis between plasma homocysteine level and polymorphism of homocysteine metabolism related enzymes in ischemic cerebrovascular or cardiovascular diseases. Zhonghua Xue Ye Xue Za Zhi.

[123] Chwatko G, Boers GH, Strauss KA (2007). Mutations in methylenetetrahydrofolate reductase or cystathionine beta-synthase gene, or a high-methionine diet, increase homocysteine thiolactone levels in humans and mice. FASEB J.

[124] Harker LA, Slichter SJ, Scott CR (1974). Homocystinemia. Vascular injury and arterial thrombosis. N Engl J Med.

[125] Endo N, Nishiyama K, Otsuka A (2006). Antioxidant activity of vitamin B6 delays homocysteine-induced atherosclerosis in rats. Br J Nutr.

[126] Maestro de las Casas C, Epeldegui M, Tudela C (2003). High exogenous homocysteine modifies eye development in early chick embryos. Birth Defects Res A Clin Mol Teratol.

[127] Li Y, Zhao Q, Liu XL (2008). Relationship between cystathionine gamma-lyase gene polymorphism and essential hypertension in Northern Chinese Han population. Chin Med J (Engl).

[128] Wang J, Huff AM, Spence JD (2004). Single nucleotide polymorphism in CTH associated with variation in plasma homocysteine concentration. Clin Genet.

[129] Mrozikiewicz PM, Bogacz A, Omielańczyk M (2015). The importance of rs1021737 and rs482843 polymorphisms of cystathionine gamma-lyase in the etiology of preeclampsia in the Caucasian population. Ginekol Pol.

[130] Zhu W, Lin A, Banerjee R (2008). Kinetic properties of polymorphic variants and pathogenic mutants in human cystathionine gamma-lyase. Biochemistry.

[131] Billaut-Laden I, Rat E, Allorge D (2006). Evidence for a functional genetic polymorphism of the human mercaptopyruvate sulfurtransferase (MPST), a cyanide detoxification enzyme. Toxicol Lett.

[132] Eto K, Kimura H (2002). A novel enhancing mechanism for hydrogen sulfide-producing activity of cystathionine beta-synthase. J Biol Chem.

[133] Ereño-Orbea J, Majtan T, Oyenarte I (2014). Structural insight into the molecular mechanism of allosteric activation of human cystathionine β-synthase by *S*-adenosylmethionine. Proc Natl Acad Sci USA.

[134] Huang S, Chua JH, Yew WS (2010). Site-directed mutagenesis on human cystathionine-gamma-lyase reveals insights into the modulation of H_2_S production. J Mol Biol.

[135] Tang XQ, Chen RQ, Ren YK (2011). ACS6, a Hydrogen sulfide-donating derivative of sildenafil, inhibits homocysteine-induced apoptosis by preservation of mitochondrial function. Med Gas Res.

[136] Tang XQ, Chen RQ, Dong L (2013). Role of paraoxonase-1 in the protection of hydrogen sulfide-donating sildenafil (ACS6) against homocysteine-induced neurotoxicity. J Mol Neurosci.

[137] Sen U, Sathnur PB, Kundu S (2012). Increased endogenous H_2_S generation by CBS, CSE, and 3MST gene therapy improves ex vivo renovascular relaxation in hyperhomocysteinemia. Am J Physiol Cell Physiol.

[138] Pushpakumar S, Kundu S, Sen U (2014). Endothelial dysfunction: the link between homocysteine and hydrogen sulfide. Curr Med Chem.

[139] Chang L, Geng B, Yu F (2008). Hydrogen sulfide inhibits myocardial injury induced by homocysteine in rats. Amino Acids.

[140] Wang R (2009). Hydrogen sulfide: a new EDRF. Kidney Int.

[141] Pan LL, Liu XH, Gong QH (2011). Hydrogen sulfide attenuated tumor necrosis factor-α-induced inflammatory signaling and dysfunction in vascular endothelial cells. PLoS One.

[142] Li L, Whiteman M, Guan YY (2008). Characterization of a novel, water-soluble hydrogen sulfide-releasing molecule (GYY4137): new insights into the biology of hydrogen sulfide. Circulation.

[143] Lynn EG, Austin RC (2011). Hydrogen sulfide in the pathogenesis of atherosclerosis and its therapeutic potential. Expert Rev Clin Pharmacol.

[144] Meng QH, Yang G, Yang W (2007). Protective effect of hydrogen sulfide on balloon injury-induced neointima hyperplasia in rat carotid arteries. Am J Pathol.

[145] Yang G, Li H, Tang G (2012). Increased neointimal formation in cystathionine gamma-lyase deficient mice: role of hydrogen sulfide in α5β1-integrin and matrix metalloproteinase-2 expression in smooth muscle cells. J Mol Cell Cardiol.

[146] Yang G, Wu L, Wang R (2006). Pro-apoptotic effect of endogenous H_2_S on human aorta smooth muscle cells. FASEB J.

[147] Zhao ZZ, Wang Z, Li GH (2011). Hydrogen sulfide inhibits macrophage-derived foam cell formation. Exp Biol Med (Maywood).

[148] Zhang H, Guo C, Wu D (2012). Hydrogen sulfide inhibits the development of atherosclerosis with suppressing CX3CR1 and CX3CL1 expression. PLoS One.

[149] Wang XH, Wang F, You SJ (2013). Dysregulation of cystathionine γ-lyase (CSE)/hydrogen sulfide pathway contributes to ox-LDL-induced inflammation in macrophage. Cell Signal.

[150] Wu SY, Pan CS, Geng B (2006). Hydrogen sulfide ameliorates vascular calcification induced by vitamin D3 plus nicotine in rats. Acta Pharmacol Sin.

[151] Zagli G, Patacchini R, Trevisani M (2007). Hydrogen sulfide inhibits human platelet aggregation. Eur J Pharmacol.

[152] Grambow E, Mueller-Graf F, Delyagina E (2007). Effect of the hydrogen sulfide donor GYY4137 on platelet activation and microvascular thrombus formation in mice. Platelets.

[153] Qiao W, Chaoshu T, Hongfang J (2010). Endogenous hydrogen sulfide is involved in the pathogenesis of atherosclerosis. Biochem Biophys Res Commun.

[154] Xu S, Liu Z, Liu P (2014). Targeting hydrogen sulfide as a promising therapeutic strategy for atherosclerosis. Int J Cardiol.

[155] Wang W, Feng SJ, Li H (2015). Correlation of lower concentrations of hydrogen sulfide with activation of protein kinase CβII in uremic accelerated atherosclerosis patients. Chin Med J.

[156] Li H, Feng SJ, Zhang GZ (2015). Correlation of lower concentrations of hydrogen sulfide with atherosclerosis in chronic hemodialysis patients with diabetic nephropathy. Blood Purif.

[157] Jiang HL, Wu HC, Li ZL (2005). Changes of the new gaseous transmitter H_2_S in patients with coronary heart disease. Di Yi Jun Yi Da Xue Xue Bao..

[158] Chen L, Ingrid S, Ding YG (2007). Imbalance of endogenous homocysteine and hydrogen sulfide metabolic pathway in essential hypertensive children. Chin Med J (Engl)..

[159] Perna AF, Luciano M, Ingrosso D (2009). Hydrogen sulphide-generating pathways in haemodialysis patients: a study on relevant metabolites and transcriptional regulation of genes encoding for key enzymes. Nephrol Dial Transplant.

[160] Perna AF, Di Nunzio A, Amoresano A (2016). Divergent behavior of hydrogen sulfide pools and of the sulfur metabolite lanthionine, a novel uremic toxin, in dialysis patients. Biochimie.

[161] Li JJ, Li Q, Du HP (2015). Homocysteine triggers inflammatory responses in macrophages through inhibiting CSE-H_2_S signaling via DNA hypermethylation of CSE promoter. Int J Mol Sci.

[162] Xu Y, Du HP, Li J (2014). Statins upregulate cystathionine γ-lyase transcription and H_2_S generation via activating Akt signaling in macrophage. Pharmacol Res.

[163] Yang G, Cao K, Wu L (2004). Cystathionine gamma-lyase overexpression inhibits cell proliferation via a H_2_S-dependent modulation of ERK1/2 phosphorylation and p21Cip/WAK-1. J Biol Chem.

[164] Shen Y, Shen Z, Miao L (2015). miRNA-30 family inhibition protects against cardiac ischemic injury by regulating cystathionine-γ-lyase expression. Antioxid Redox Signal.

[165] Fox B, Schantz JT, Haigh R (2012). Inducible hydrogen sulfide synthesis in chondrocytes and mesenchymal progenitor cells: is H_2_S a novel cytoprotective mediator in the inflamed joint?. J Cell Mol Med.

[166] Badiei A, Rivers-Auty J, Ang AD (2013). Inhibition of hydrogen sulfide production by gene silencing attenuates inflammatory activity of LPS-activated RAW264.7 cells. Appl Microbiol Biotechnol.

[167] Badiei A, Muniraj N, Chambers S (2014). Inhibition of hydrogen sulfide production by gene silencing attenuates inflammatory activity by downregulation of NF-κB and MAP kinase activity in LPS-activated RAW 264.7 cells. Biomed Res.

[168] Wang K, Ahmad S, Cai M (2013). Dysregulation of hydrogen sulfide producing enzyme cystathionine γ-lyase contributes to maternal hypertension and placental abnormalities in preeclampsia. Circulation.

[169] Liu Y, Yang R, Liu X (2014). Hydrogen sulfide maintains mesenchymal stem cell function and bone homeostasis via regulation of Ca(^2+^) channel sulfhydration. Cell Stem Cell.

[170] Sen U, Givvimani S, Abe OA (2007). Cystathionine β-synthase and cystathionine γ-lyase double gene transfer ameliorate homocysteine-mediated mesangial inflammation through hydrogen sulfide generation. Am J Physiol Cell Physiol.

[171] Albertini E, Kozieł R, Dürr A (2012). Cystathionine beta synthase modulates senescence of human endothelial cells. Aging (Albany NY).

[172] Bhattacharyya S, Saha S, Giri K (2013). Cystathionine beta-synthase (CBS) contributes to advanced ovarian cancer progression and drug resistance. PLoS One.

[173] Szabo C, Coletta C, Chao C (2013). Tumor-derived hydrogen sulfide, produced by cystathionine-β-synthase, stimulates bioenergetics, cell proliferation, and angiogenesis in colon cancer. Proc Natl Acad Sci USA.

[174] Miller TW, Wang EA, Gould S (2011). Hydrogen sulfide is an endogenous potentiator of T cell activation. J Biol Chem.

[175] Sen S, Kawahara B, Gupta D (2015). Role of cystathionine β-synthase in human breast Cancer. Free Radic Biol Med.

[176] Yang G, Yang W, Wu L (2007). H_2_S, endoplasmic reticulum stress, and apoptosis of insulin-secreting beta cells. J Biol Chem.

[177] Watanabe M, Osada J, Aratani Y (1995). Mice deficient in cystathionine beta-synthase: animal models for mild and severe homocyst(e)inemia. Proc Natl Acad Sci USA.

[178] Wang L, Jhee KH, Hua X (2004). Modulation of cystathionine beta-synthase level regulates total serum homocysteine in mice. Circ Res.

[179] Wang L, Chen X, Tang B (2005). Expression of mutant human cystathionine beta-synthase rescues neonatal lethality but not homocystinuria in a mouse model. Hum Mol Genet.

[180] Maclean KN, Sikora J, Kožich V (2010). A novel transgenic mouse model of CBS-deficient homocystinuria does not incur hepatic steatosis or fibrosis and exhibits a hypercoagulative phenotype that is ameliorated by betaine treatment. Mol Genet Metab.

[181] Lentz SR, Erger RA, Dayal S (2000). Folate dependence of hyperhomocysteinemia and vascular dysfunction in cystathionine beta-synthase-deficient mice. Am J Physiol Heart Circ Physiol.

[182] Clarke ZL, Moat SJ, Miller AL (2006). Differential effects of low and high dose folic acid on endothelial dysfunction in a murine model of mild hyperhomocysteinaemia. Eur J Pharmacol.

[183] Kalani A, Kamat PK, Familtseva A (2014). Role of microRNA29b in blood–brain barrier dysfunction during hyperhomocysteinemia: an epigenetic mechanism. J Cereb Blood Flow Metab.

[184] Dayal S, Bottiglieri T, Arning E (2001). Endothelial dysfunction and elevation of S-adenosylhomocysteine in cystathionine beta-synthase-deficient mice. Circ Res.

[185] Dayal S, Arning E, Bottiglieri T (2004). Cerebral vascular dysfunction mediated by superoxide in hyperhomocysteinemic mice. Stroke.

[186] Robert K, Santiard-Baron D, Chassé JF (2004). The neuronal SAPK/JNK pathway is altered in a murine model of hyperhomocysteinemia. J Neurochem.

[187] Vitvisky V, Dayal S, Stabler S (2004). Perturbations in homocysteine-linked redox homeostasis in a murine model for hyperhomocysteinemia. Am J Physiol Regul Integr Comp Physiol.

[188] Kundu S, Kumar M, Sen U (2009). Nitrotyrosinylation, remodeling and endothelial-myocyte uncoupling in iNOS, cystathionine beta synthase (CBS) knockouts and iNOS/CBS double knockout mice. J Cell Biochem.

[189] Mayo JN, Beard RS, Price TO (2012). Nitrative stress in cerebral endothelium is mediated by mGluR5 in hyperhomocysteinemia. J Cereb Blood Flow Metab.

[190] Shastry S, Moning L, Tyagi N (2005). GABA receptors and nitric oxide ameliorate constrictive collagen remodeling in hyperhomocysteinemia. J Cell Physiol.

[191] Kamath AF, Chauhan AK, Kisucka J (2006). Elevated levels of homocysteine compromise blood–brain barrier integrity in mice. Blood.

[192] Hamelet J, Maurin N, Fulchiron R (2007). Mice lacking cystathionine beta synthase have lung fibrosis and air space enlargement. Exp Mol Pathol.

[193] Hamelet J, Demuth K, Paul JL (2007). Hyperhomocysteinemia due to cystathionine beta synthase deficiency induces dysregulation of genes involved in hepatic lipid homeostasis in mice. J Hepatol.

[194] Liao D, Tan H, Hui R (2006). Hyperhomocysteinemia decreases circulating high-density lipoprotein by inhibiting apolipoprotein A-I Protein synthesis and enhancing HDL cholesterol clearance. Circ Res.

[195] Gupta S, Kruger WD (2011). Cystathionine beta-synthase deficiency causes fat loss in mice. PLoS One.

[196] Ganapathy PS, Moister B, Roon P (2009). Endogenous elevation of homocysteine induces retinal neuron death in the cystathionine-beta-synthase mutant mouse. Invest Ophthalmol Vis Sci.

[197] Nuño-Ayala M, Guillén N, Arnal C (2012). Cystathionine β-synthase deficiency causes infertility by impairing decidualization and gene expression networks in uterus implantation sites. Physiol Genomics.

[198] Guzman MA, Navarro MA, Carnicer R (2006). Cystathionine beta-synthase is essential for female reproductive function. Hum Mol Genet.

[199] Hagiya Y, Kamata S, Mitsuoka S (2015). Hemizygosity of transsulfuration genes confers increased vulnerability against acetaminophen-induced hepatotoxicity in mice. Toxicol Appl Pharmacol.

[200] Okomoto M, Yamaoka M, Takei M (2013). Endogenous hydrogen sulfide protects pancreatic beta-cells from a high-fat diet-induced glucotoxicity and prevents the development of type 2 diabetes. Biochem Biophys Res Commun..

[201] Tang G, Zhang L, Yang G (2013). Hydrogen sulfide-induced inhibition of L-type Ca^2+^ channels and insulin secretion in mouse pancreatic beta cells. Diabetologia.

[202] King AL, Polhemus DJ, Bhushan S (2015). Hydrogen sulfide cytoprotective signaling is endothelial nitric oxide synthase-nitric oxide dependent. Proc Natl Acad Sci USA.

[203] Ishii I, Akahoshi N, Yamada H (2010). Cystathionine gamma-lyase-deficient mice require dietary cysteine to protect against acute lethal myopathy and oxidative injury. J Biol Chem.

[204] Yang G, Wu L, Jiang B (2008). H_2_S as a physiologic vasorelaxant: hypertension in mice with deletion of cystathionine gamma-lyase. Science.

[205] Yang G, Wu L, Bryan S (2010). Cystathionine gamma-lyase deficiency and overproliferation of smooth muscle cells. Cardiovasc Res.

[206] Peng YJ, Nanduri J, Raghuraman G (2010). H_2_S mediates O_2_ sensing in the carotid body. Proc Natl Acad Sci USA.

[207] Yang G, Zhao K, Ju Y (2013). Hydrogen sulfide protects against cellular senescence via S-sulfhydration of Keap1 and activation of Nrf2. Antioxid Redox Signal.

[208] Kondo K, Bhushan S, King AL (2013). H_2_S protects against pressure overload-induced heart failure via upregulation of endothelial nitric oxide synthase. Circulation.

[209] Zhang G, Wang P, Yang G (2013). The inhibitory role of hydrogen sulfide in airway hyperresponsiveness and inflammation in a mouse model of asthma. Am J Pathol.

[210] Tang G, Yang G, Jiang B (2013). H_2_S is an endothelium-derived hyperpolarizing factor. Antioxid Redox Signal.

[211] Bos EM, Wang R, Snijder PM (2013). Cystathionine γ-lyase protects against renal ischemia/reperfusion by modulating oxidative stress. J Am Soc Nephrol.

[212] Mani S, Li H, Untereiner A (2013). Decreased endogenous production of hydrogen sulfide accelerates atherosclerosis. Circulation.

[213] Ang AD, Rivers-Auty J, Hegde A (2013). The effect of CSE gene deletion in caerulein-induced acute pancreatitis in the mouse. Am J Physiol Gastrointest Liver Physiol.

[214] Jiang Z, Li C, Manuel ML (2015). Role of hydrogen sulfide in early blood–brain barrier disruption following transient focal cerebral ischemia. PLoS One.

[215] Kolluru GK, Bir SC, Yuan S (2015). Cystathionine γ-lyase regulates arteriogenesis through NO-dependent monocyte recruitment. Cardiovasc Res.

[216] Syhr KM, Boosen M, Hohmann SW (2015). The H_2_S-producing enzyme CSE is dispensable for the processing of inflammatory and neuropathic pain. Brain Res.

[217] Untereiner AA, Wang R, Ju Y (2016). Decreased Gluconeogenesis in the Absence of Cystathionine Gamma-Lyase and the Underlying Mechanisms. Antioxid Redox Signal.

[218] Ivanciuc T, Sbrana E, Ansar M (2016). Hydrogen sulfide: an antiviral and anti-inflammatory endogenous gasotransmitter in the airways. Role in respiratory syncytial virus infection. Am J Respir Cell Mol Biol.

[219] Miao L, Xin X, Xin H (2016). Hydrogen sulfide recruits macrophage migration by integrin β1-Src-FAK/Pyk2-Rac pathway in myocardial infarction. Sci Rep.

[220] Ali MY, Ping CY, Mok YY (2006). Regulation of vascular nitric oxide in vitro and in vivo; a new role for endogenous hydrogen sulphide?. Br J Pharmacol.

[221] Filipovic MR, Miljkovic JL, Nauser T (2012). Chemical characterization of the smallest S-nitrosothiol, HSNO; cellular cross-talk of H_2_S and S-nitrosothiols. J Am Chem Soc.

[222] Cortese-Krott MM, Fernandez BO, Santos JL (2014). Nitrosopersulfide (SSNO(-)) accounts for sustained NO bioactivity of S-nitrosothiols following reaction with sulfide. Redox Biol.

[223] Cheung SH, Kwok WK, To KF (2014). Anti-atherogenic effect of hydrogen sulfide by over-expression of cystathionine gamma-lyase (CSE) gene. PLoS One.

[224] Kimura Y, Toyofuku Y, Koike S (2015). Identification of H_2_S_3_ and H_2_S produced by 3-mercaptopyruvate sulfurtransferase in the brain. Sci Rep.

[225] Yadav PK, Yamada K, Chiku T (2013). Structure and kinetic analysis of H2S production by human mercaptopyruvate sulfurtransferase. J Biol Chem.

[226] Nicholls P (1975). Inhibition of cytochrome c oxidase by sulphide. Biochem Soc Trans.

[227] Nagahara N, Nagano M, Ito T (2013). Antioxidant enzyme, 3-mercaptopyruvate sulfurtransferase-knockout mice exhibit increased anxiety-like behaviors: a model for human mercaptolactate-cysteine disulfiduria. Sci Rep.

[228] Vande Weghe JG, Ow DW (1999). A fission yeast gene for mitochondrial sulfide oxidation. J Biol Chem.

[229] Libiad M, Yadav PK, Vitvitsky V (2014). Organization of the human mitochondrial hydrogen sulfide oxidation pathway. J Biol Chem.

[230] Ackermann M, Kubitza M, Hauska G (2014). The vertebrate homologue of sulfide-quinone reductase in mammalian mitochondria. Cell Tissue Res.

[231] Olson KR (2015). Hydrogen sulfide as an oxygen sensor. Antioxid Redox Signal.

[232] Jin HS, Kim J, Park S (2016). Association of the I264T variant in the sulfide quinone reductase-like (SQRDL) gene with osteoporosis in Korean postmenopausal women. PLoS One.

[233] Croteau-Chonka DC, Marvelle AF, Lange EM (2011). Genome-wide association study of anthropometric traits and evidence of interactions with age and study year in Filipino women. Obesity (Silver Spring).

[234] Itou T, Maldonado N, Yamada I (2014). Cystathionine gamma-lyase accelerates osteoclast differentiation: identification of a novel regulator of osteoclastogenesis by proteomic analysis. Arterioscler Thromb Vasc Biol.

[235] Gambari L, Lisignoli G, Cattini L (2014). Sodium hydrosulfide inhibits the differentiation of osteoclast progenitor cells via NRF2-dependent mechanism. Pharmacol Res.

[236] Pan Z, Wang H, Liu Y (2014). Involvement of CSE/H_2_S in high glucose induced aberrant secretion of adipokines in 3T3-L1 adipocytes. Lipids Health Dis.

[237] Geng B, Cai B, Liao F (2013). Increase or decrease hydrogen sulfide exert opposite lipolysis, but reduce global insulin resistance in high fatty diet induced obese mice. PLoS One.

[238] Carter RN, Morton NM (2016). Cysteine and hydrogen sulphide in the regulation of metabolism: insights from genetics and pharmacology. J Pathol.

[239] Jain S, Micinski D, Lieblong BJ (2012). Relationship between hydrogen sulfide levels and HDL-cholesterol, adiponectin, and potassium levels in the blood of healthy subjects. Atherosclerosis.

[240] Liang M, Jin S, Wu DD (2015). Hydrogen sulfide improves glucose metabolism and prevents hypertrophy in cardiomyocytes. Nitric Oxide.

[241] Horsman JW, Miller DL (2016). Mitochondrial sulfide quinone oxidoreductase prevents activation of the unfolded protein response in hydrogen sulfide. J Biol Chem.

[242] Henriques BJ, Lucas TG, Rodrigues JV (2014). Ethylmalonic encephalopathy ETHE1 R163W/R163Q mutations alter protein stability and redox properties of the iron centre. PLoS One.

[243] Zafeiriou DI, Augoustides-Savvopoulou P, Haas D (2007). Ethylmalonic encephalopathy: clinical and biochemical observations. Neuropediatrics.

[244] Lagoutte E, Mimoun S, Andriamihaja M (2010). Oxidation of hydrogen sulfide remains a priority in mammalian cells and causes reverse electron transfer in colonocytes. Biochim Biophys Acta.

[245] Hildbrant TM, Di Meo I, Zeviani M (2013). Proteome adaptations in Ethe1-deficient mice indicate a role in lipid catabolism and cytoskeleton organization via post-translational protein modifications. Biosci Rep.

[246] Ono K, Akaike T, Sawa T (2014). Redox chemistry and chemical biology of H_2_S, hydropersulfides, and derived species: implications of their possible biological activity and utility. Free Radic Biol Med.

[247] Jain SK, Bull R, Rains JL (2010). Low levels of hydrogen sulfide in the blood of diabetes patients and streptozotocin-treated rats causes vascular inflammation?. Antioxid Redox Signal.

[248] Baiges I, Palmfeldt J, Bladé C (2010). Lipogenesis is decreased by grape seed proanthocyanidins according to liver proteomics of rats fed a high fat diet. Mol Cell Proteomics.

[249] Jurkowska H, Roman HB, Hirschberger LL (2014). Primary hepatocytes from mice lacking cysteine dioxygenase show increased cysteine concentrations and higher rates of metabolism of cysteine to hydrogen sulfide and thiosulfate. Amino Acids.

[250] Roman HB, Hirschberger LL, Krijt J (2013). The cysteine dioxgenase knockout mouse: altered cysteine metabolism in nonhepatic tissues leads to excess H_2_S/HS(–) production and evidence of pancreatic and lung toxicity. Antioxid Redox Signal.

[251] Ueki I, Roman HB, Valli A (2011). Knockout of the murine cysteine dioxygenase gene results in severe impairment in ability to synthesize taurine and an increased catabolism of cysteine to hydrogen sulfide. Am J Physiol Endocrinol Metab.

[252] Weiss N, Heydrick S, Zhang YY (2002). Cellular redox state and endothelial dysfunction in mildly hyperhomocysteinemic cystathionine b-synthase-deficient mice. Arterioscler Thromb Vasc Biol.

[253] Flannigan KL, Ferraz JG, Wang R (2013). Enhanced synthesis and diminished degradation of hydrogen sulfide in experimental colitis: a site-specific, pro-resolution mechanism. PLoS One.

[254] Madurga A, Golec A, Pozarska A (2015). The H_2_S-generating enzymes cystathionine β-synthase and cystathionine γ-lyase play a role in vascular development during normal lung alveolarization. Am J Physiol Lung Cell Mol Physiol.

[255] Sen U, Basu P, Abe OA (2009). Hydrogen sulfide ameliorates hyperhomocysteinemia-associated chronic renal failure. Am J Physiol Renal Physiol.

[256] Módis K, Wolanska K, Vozdek R (2013). Hydrogen sulfide in cell signaling, signal transduction, cellular bioenergetics and physiology in *C. elegans*. Gen Physiol Biophys.

[257] Calderwood A, Kopriva S (2014). Hydrogen sulfide in plants: from dissipation of excess sulfur to signaling molecule. Nitric Oxide.

[258] Miller DL, Roth MB (2007). Hydrogen sulfide increases thermotolerance and lifespan in *Caenorhabditis elegans*. Proc Natl Acad Sci USA.

[259] Budde MW, Roth MB (2011). The response of *Caenorhabditis elegans* to hydrogen sulfide and hydrogen cyanide. Genetics.

[260] Hine C, Harputlugil E, Zhang Y (2015). Endogenous hydrogen sulfide production is essential for dietary restriction benefits. Cell.

[261] Hine C, Mitchell JR (2015). Calorie restriction and methionine restriction in control of endogenous hydrogen sulfide production by the transsulfuration pathway. Exp Gerontol.

[262] Kabil H, Kabil O, Banerjee R (2011). Increased transsulfuration mediates longevity and dietary restriction in Drosophila. Proc Natl Acad Sci USA.

[263] Snijder PM, Baratashvili M, Grzeschik NA (2015). Overexpression of cystathionine γ-lyase suppresses detrimental effects of spinocerebellar ataxia type 3. Mol Med.

[264] Kumai Y, Porteus CS, Kwong RW (2015). Hydrogen sulfide inhibits Na^+^ uptake in larval zebrafish, *Danio rerio*. Pflugers Arch.

[265] Laubach VE, Shesely EG, Smithies O (1995). Mice lacking inducible nitric oxide synthase are not resistant to lipopolysaccharide-induced death. Proc Natl Acad Sci USA.

[266] Shesely EG, Maeda N, Kim HS (1996). Elevated blood pressures in mice lacking endothelial nitric oxide synthase. Proc Natl Acad Sci USA.

[267] Duplain H, Burcelin R, Sartori C (2001). Insulin resistance, hyperlipidemia, and hypertension in mice lacking endothelial nitric oxide synthase. Circulation.

[268] Kodela R, Chattopadhyay M, Kashfi K (2012). NOSH-aspirin: a novel nitric oxide-hydrogen sulfide-releasing hybrid: a new class of anti-inflammatory pharmaceuticals. ACS Med Chem Lett.

[269] Fonseca MD, Cunha FQ, Kashfi K (2015). NOSH-aspirin (NBS-1120), a dual nitric oxide and hydrogen sulfide-releasing hybrid, reduces inflammatory pain. Pharmacol Res Perspect.

[270] Hu Q, Wu D, Ma F (2016). Novel angiogenic activity and molecular mechanisms of ZYZ-803, a slow-releasing hydrogen sulfide-nitric oxide hybrid molecule. Antioxid Redox Signal.

